# Ensemble Learning Framework for Anomaly Detection in Autonomous Driving Systems

**DOI:** 10.3390/s25165105

**Published:** 2025-08-17

**Authors:** Sazid Nazat, Walaa Alayed, Lingxi Li, Mustafa Abdallah

**Affiliations:** 1 Elmore Family School of Electrical and Computer Engineering, Purdue University in Indianpolis, Indianapolis, IN 46202, USA; snazat@purdue.edu (S.N.); lingxili@purdue.edu (L.L.); 2 Department of Information Technology, College of Computer and Information Sciences, Princess Nourah Bint Abdulrahman University, Riyadh 11671, Saudi Arabia; 3 Computer and Information Technology Department, Purdue University in Indianapolis, Indianapolis, IN 46202, USA; abdalla0@purdue.edu

**Keywords:** anomaly detection, ensemble learning, autonomous driving systems, VANET security, data engineering, machine learning

## Abstract

**Highlights:**

**What are the main findings?**

- Our proposed ensemble models for anomaly detection in autonomous driving systems consistently outperform individual models in all accuracy metrics on two datasets.
- In evaluating false positive rates, ensemble learning demonstrated significant gains, reducing false positives and thereby enhancing overall system reliability.

**What is the implication of the main finding?**

- The findings underscore the efficacy of ensemble learning in enhancing resilience and precision of anomaly detection systems in autonomous vehicles.
- Enhanced anomaly detection bolsters public confidence in autonomous driving systems.

**Abstract:**

The inherent limitations of individual AI models underscore the need for robust anomaly detection techniques for securing autonomous driving systems. To address these limitations, we propose a comprehensive ensemble learning framework specifically designed for anomaly detection in autonomous driving systems. We comprehensively assess the effectiveness of ensemble learning models for detecting anomalies in autonomous vehicle datasets, focusing primarily on the VeReMi and Sensor datasets. Ensemble techniques are rigorously evaluated against individual models on binary and multiclass classification tasks. The analysis reveals that ensemble models consistently outperform individual models in terms of accuracy, precision, recall, false positive rates, and F1-score. On the VeReMi dataset, ensembles achieve high performance for binary classification, with a maximum accuracy of 0.80 and F1-score of 0.86, surpassing single models. For the Sensor dataset, ensemble models like CatBoost exhibit perfect accuracy, precision, recall, and F1-score, exceeding single models by 11% in accuracy. In VeReMi multiclass classification, Stacking and Blending gave a 5% increase in accuracy compared to single models. Moreover, XGBoost and CatBoost demonstrate perfect recall. Our proposed method enhanced performance despite the increased runtime required by ensemble models. In evaluating false positive rates, ensemble learning demonstrated significant gains, reducing false positives and thereby enhancing overall system reliability.

## 1. Introduction

The proliferation of autonomous vehicles (AVs) and vehicular ad hoc networks (VANETs) (note that VANETs are a specialized form of mobile ad hoc networks designed to enable communication between vehicles and between vehicles and roadside infrastructure) has ushered in a new era of transportation, offering enhanced safety, efficiency, and convenience. However, the intricate and interconnected nature of these systems introduces significant security vulnerabilities that, if exploited, could have catastrophic consequences [1]. Anomaly detection, a crucial aspect of cybersecurity, plays a pivotal role in safeguarding AVs and VANETs against malicious activities, whether initiated by internal users or external adversaries [2].

Traditional approaches to anomaly detection often rely on single-classifier models, such as Decision Trees, neural networks, or Support Vector Machines [3,4,5]. While these methods have demonstrated efficacy in certain contexts, they frequently struggle to capture the complexities and nuances inherent in intricate datasets associated with autonomous driving environments [6]. The inherent limitations of individual models, including high false positive rates, overfitting tendencies, and an inability for generalization, lead to the need for more robust and sophisticated anomaly detection techniques [7,8].

Ensemble learning, a paradigm that collectively combines multiple models to harness their collective strengths while mitigating individual weaknesses, has emerged as a promising solution to enhance anomaly detection capabilities [9]. By integrating diverse algorithms, such as bagging, boosting, stacking, and blending, ensemble methods leverage the complementary strengths of constituent models, potentially leading to improved predictive performance, heightened generalization abilities, and increased robustness against noise and outliers. Although ensemble learning has been applied extensively for different applications (see [10] for a survey), few works leveraged ensemble learning techniques for enhancing AI-based anomaly detection for autonomous driving systems [11,12,13].

Prior work [13] has implemented an ensemble approach using CNN-based regression models for autonomous driving on image and steering angle data to minimize the risks associated with unsafe driving. Du et al.’s work [14] demonstrates how spatio-temporal convolutional networks can predict future LiDAR frames without labeled data, which aligns with the broader trend of reducing reliance on manual annotations while improving foresight in AV systems. The study [11] utilized a semi-supervised approach using K-nearest Neighbors (K-NN)-based ensemble learning for classifying the maneuvering behaviors of surrounding vehicles in autonomous driving scenarios. The authors in [12] utilized an ensemble learning-based method for recognizing aggressive driving behavior (ADB) to address the limitations of previous data-driven approaches employing three deep learning methods (CNN, LSTM, and GRU) as base classifiers, and combining them using various rules to form ensemble classifiers. Our work distinguishes itself from prior studies [11,12,13] through its comprehensive scope and analytical depth. While previous research focused on specific aspects of autonomous driving, such as steering angle prediction or behavior recognition, we present a holistic ensemble learning framework for anomaly detection across both autonomous driving systems and VANETs. Our approach is more expansive, exploring 11 diverse ensemble techniques compared to the limited ensemble methods in these earlier works. We conduct rigorous evaluations on two distinct real-world datasets, offering a broader perspective than the specialized datasets used previously. Unlike prior studies, we provide an in-depth comparative analysis between ensemble methods and single-classifier models, coupled with detailed insights into their strengths, limitations, and trade-offs specific to AV anomaly detection. Our focus on improved generalization and the provision of open-source code further sets our work apart, fostering broader applicability and future development in the field. This comprehensive approach enables a more robust and versatile framework for addressing the complex challenges of anomaly detection in autonomous driving environments.

Motivated by the pressing need to fortify the security of intelligent transportation systems, this paper proposes a comprehensive comparison between single (individual) learning models (we use “single” and “individual” interchangeably throughout the paper to indicate non-ensemble learning AI models) and ensemble learning framework tailored explicitly for anomaly detection in autonomous driving systems. Our framework harnesses a diverse array of ensemble techniques, adaptive boosting [15], gradient boosting machines [15], and stacking ensembles [16]. Through this multi-faceted approach, we aim to achieve superior predictive performance, improved generalization, and heightened robustness compared to traditional single-classifier models.

To rigorously evaluate the efficacy of our proposed framework, we employ two distinct real-world datasets: the VeReMi dataset [17] and the Sensor dataset [2,18]. The VeReMi dataset encompasses vehicle telemetry data and various types of attacks, such as denial of service, Sybil attacks, and message falsification in VANETs [19]. Conversely, the Sensor dataset comprises simulated sensor measurements from AVs, capturing potential anomalies across multiple dimensions, including speed, lane positioning, and communication protocol adherence. Our evaluation shows that the proposed ensemble learning framework demonstrates the effectiveness of combining multiple machine learning models for enhanced anomaly detection in autonomous vehicle datasets. The ensemble methods, such as Random Forest, Bagging, and Stacking, consistently outperformed single classifiers across various evaluation metrics, including accuracy, precision, recall, and F1-score. The ensemble techniques leverage the strengths of individual models while mitigating their weaknesses, resulting in improved robustness and generalization capabilities. We obtain an 11% boost in accuracy over single classifiers using ensemble learning models, demonstrating their superiority. Additionally, the analysis reveals the trade-offs between different hyperparameter settings and their impact on model performance, highlighting the importance of careful hyperparameter tuning. Furthermore, this study provides insights into the computational efficiency of the evaluated models and identifies the most time-efficient approaches for practical deployments. We also discuss potential optimizations for enhancing runtime performance of advanced ensemble methods for such deployments. Our evaluation also shows that the ensemble learning methods lead to an increase in false positive rates for both datasets. We also provide feature importance analysis for both single and ensemble methods.

Ultimately, this research endeavors to address the vulnerabilities inherent in AVs and VANETs, paving the way for their safe and widespread adoption. By fortifying these systems against malicious attacks and anomalies, our work aims to bolster public confidence in autonomous driving technologies, thereby facilitating a seamless transition towards more efficient, sustainable, and intelligent transportation networks worldwide.

**Summary of Contributions:** The main contributions of this paper can be summarized as follows:

- We propose an ensemble learning framework for anomaly detection in autonomous driving systems. In our framework, we perform a comprehensive evaluation of various ensemble learning methods along with individual models.
- We employ a diverse array of ensemble techniques exploring 11 ensemble learning models, including random forest (RF), bagging classifier (bagging), adaptive boosting (AdaBoost), eXtreme Gradient Boosting (XGBoost), categorical boosting (CatBoost), light gradient boosting machine (LGBM), gradient boosting machine (GBM), average (Avg), weighted average (W. Avg), stacking (Stacking), and blending (Blending). We achieve superior predictive performance compared to single-classifier models.
- We conduct a rigorous evaluation of the proposed ensemble framework using two distinct real-world autonomous driving datasets with different characteristics—the VeReMi and Sensor datasets.
- We provide insights into strengths, limitations, and trade-offs of different ensemble learning methods for the anomaly detection task for autonomous vehicles.
- We release our source codes to facilitate their use in autonomous driving anomaly detection classification. We emphasize that this resource has the two datasets, preprocessing scripts, and hyperparameter tuning settings to facilitate full reproducibility of our results. We encourage researchers to utilize this resource for further development and to build additional models (the URL for our source codes can be found at https://github.com/Nazat28/Ensemble-Models-for-Classification-on-Autonomous-Vehicle-Dataset, accessed on 1 August 2025).

The rest of the paper is structured as follows: Section 2 covers related work, while Section 3 presents the background and problem statement. Section 4 details our ensemble learning framework. Next, Section 5 and Section 6 analyze the framework’s performance and model efficiency. Section 7 explores limitations and broader implications, and Section 8 concludes with key findings and future research directions.

## 2. Related Work

### 2.1. Anomaly Detection in AV

Multiple studies have investigated the detection of anomalies in autonomous driving systems, as evidenced by the works [20,21,22,23]. The authors employed a modified-convolutional neural network (M-CNN) to analyze both onboard and exterior sensor readings, as stated in article [20]. The sensor data refers to the information collected by the sensors in the autonomous vehicle. In this case, the characteristics extracted from the sensor data include the vehicle speed, GPS speed, and vehicle acceleration. These features are used to identify sudden abnormalities in the autonomous vehicle. Anomaly detection is the identification of unexpected and significant fluctuations in sensor data values or rapid shifts in GPS location coordinates. The study conducted by [21] employed convolutional neural networks (CNNs) and Kalman filtering techniques to identify anomalous behaviors in AVs. In the work [22], LSTM networks are utilized to determine false data injection (FDI) attacks, ensuring the stable operation of AVs. The authors in [23] presented a cooperative data analytics strategy to address anomalies in AVs within the network.

Several studies have explored the topic of anomaly detection in vehicle networks [23,24,25]. The study [23] employed a hybrid deep anomaly detection (HDAD) architecture, enabling AVs to identify and flag any harmful activities by analyzing shared sensor network data. Additionally, the study [24] employed a time-series anomaly detection method to identify instances of cyber intrusions or malfunctioning sensors. In prior work, ref. [25] used a CNN-based LSTM model to classify AV signals as anomalous or normal. Our framework presents a more powerful and robust approach for identifying anomalies in AVs by incorporating ensemble learning methods to secure the VANET. In the domain of autonomous systems, [26] proposes integrating Scenario Engineering with autonomous transportation systems to improve trustworthiness and efficiency in open-pit mines.

The work [27] explores the potential of generative AI models like Sora for scenario generation in Scenario Engineering for intelligent vehicles. Work [28] offers an efficient trajectory prediction model without HD maps, addressing constraints of existing approaches by storing spatial–temporal information of single and multiple agents using attention, LSTM, GCN, and transformers. Study [29] offers a reliability-based path planning method for off-road autonomous ground vehicles, using terrain uncertainty and vehicle dynamics simulations with RRT* to design optimal paths satisfying reliability constraints for mobility failure types. Work [30] covers advanced driver assistance systems to autonomous driving, including low-level feature extraction, object recognition, semantic labeling with CNNs, mapping, localization, and scene representation. In [31], the authors introduce a deep CNN-based system for recognizing and classifying seven common driver activities, including distracted behaviors such as texting and answering phones, with high accuracy using transfer learning on pre-trained CNN models such as AlexNet and ResNet50. We refer to the comprehensive review by Liang et al. [32], which surveys over 200 vehicle detection algorithms across modalities such as vision, LiDAR, radar, and sensor fusion.

However, our work complements this by evaluating diverse ensemble learning models for anomaly detection in autonomous vehicles, demonstrating their superior performance over individual models on metrics like accuracy, precision, recall, false positive rate, and F1-score, thereby enhancing the resilience and precision of such systems.

### 2.2. Ensemble Learning in Autonomous Driving

Ensemble learning has been employed in autonomous driving to increase reliability, decrease errors, improve overall performance, and ensure safety and security. Nevertheless, there is a finite quantity of literature that has addressed this topic. Paper [11] has proposed a semi-supervised ensemble approach to classify the maneuvering behaviors of surrounding vehicles in autonomous driving scenarios. The work [33] has proposed an ensemble-based machine learning framework to develop an intrusion detection system for classifying malicious and benign data requests in autonomous vehicles. Prior work [13] proposed an ensemble-based anomaly detection approach to automatically identify potential faults in vehicle data without the need for expert-defined parameters. A parallel ensemble learning approach called PelFace to enhance face-based authentication for autonomous vehicles by combining recent deep learning models with novel loss functions is proposed by work [34]. Research [35] suggested a hybrid ensemble approach that combines a random forest classifier and a deep convolutional neural network to correctly classify objects using only radar data for self-driving applications. The work [36] suggested a data-driven method based on Markov decision processes and game theory to model background vehicle behavior for autonomous vehicle testing in virtual merging scenarios.

These related works and their limitations are summarized in Table 1 where we compare our work and these previous related works. In particular, our work offers a thorough assessment of the effectiveness of ensemble learning techniques in comparison to single-classifier models on two separate datasets—VeReMi and Sensor. These datasets have different characteristics from all of those considered in the aforementioned works. In addition, we offer an examination of the performance of other ensemble strategies, such as Random Forest, Bagging, AdaBoost, XGBoost, CatBoost, LGBM, Stacking, Blending, etc., in both binary and multiclass classification problems. Furthermore, this study includes a comparative analysis of ensemble learning models and their single model counterparts (Decision Trees (DTs), K-nearest Neighbors (KNN), Support Vector Machines (SVMs), Multilayer Perceptron (MLP)). The purpose is to determine if the ensemble methods demonstrate better performance and to evaluate the computational efficiency of both the ensemble and single-classifier models on two datasets.

## 3. The Problem Statement

We now provide the main preliminaries for anomaly detection of AVs, the challenges of AI models, the need for ensemble learning, and the challenges in evaluating such methods when applied to anomaly detection in AVs.

### 3.1. Anomaly Types in AVs

There are several anomaly types in the field of autonomous vehicle networks. In our work, we consider the main six anomaly types of AVs mentioned in the VeReMi dataset [37] and Sensor dataset [2]. Thus, the anomaly types in AVs can be divided into the following categories:

**Normal:** The AV is not an attacker and behaves normally, transmitting its actual position.

**Constant:** The AV transmits a fixed, predetermined position, regardless of its actual location. This could be used to make the vehicle appear stationary or at a different location than it truly is [37].

**Constant Offset:** The AV adds a constant offset to its actual position when transmitting location data. This causes the reported position to be different from the vehicle’s real location by a fixed amount [37].

**Random:** The AV transmits a random position within the simulation area, unrelated to its true location. This can be used to make the AV appear to be moving erratically or in an unrealistic manner [37].

**Random Offset:** The AV transmits a position that is randomly offset from its actual location, with the offset coming from a pre-configured rectangular area around the AV. This can create the impression of the vehicle moving in an unpredictable way [37].

**Eventual Stop:** The AV behaves normally for some time and then repeatedly transmits its current position, effectively making it appear to have stopped moving. This could be used to simulate a vehicle that has experienced a failure or breakdown [37].

**Formality [2,18]**: The AV would be vulnerable to message spoofing and manipulation attacks. Adversaries could exploit this lack of verification to inject malformed or malicious communication packets, potentially disrupting the system’s operations and compromising the integrity of the data exchange. This could lead to incorrect decision-making, unsafe maneuvers, and a breakdown in overall coordination of the AV.

**Location [2,18]**: The AV would be susceptible to routing attacks, where communication messages are redirected to unauthorized destinations. This could enable an adversary to isolate specific AVs or gain access to sensitive information by intercepting messages meant for other components of the system. Such attacks could undermine the spatial awareness and coordination required for safe platooning operations.

**Frequency [2,18]**: Denial-of-service (DoS) attacks, in which adversaries overload the communication channels with excessive or irregular communications, could potentially compromise the AV. This might be more than the system can handle, which would cause missed deadlines, sluggish reactions, and possibly even dangerous situations.

**Speed [2,18]**: The AV would lack the ability to monitor and enforce the specified speed limits. This could enable adversaries to manipulate the AV’s speed, either by causing it to exceed the safety thresholds or by inducing sudden and unsafe speed changes. Such attacks could increase the risk of collisions, and undermine the overall safety and efficiency of the transportation network.

**Correlation [2,18]**: Some vulnerabilities target the synchronization and coherence of data across multiple communication channels which could affect the AV. This vulnerability may be exploited by adversaries to introduce discordant information, which may result in the AV network making contradictory decisions and experiencing a disruption in its coordinated behavior. Such actions may lead to hazardous maneuvers, a decline in control, and an increased probability of accidents.

**Lane Alignment [2,18]**: The AV would lack the ability to monitor and maintain the proper positioning of AVs within designated lanes. This could enable adversaries to cause individual AVs to deviate from their lanes or induce lane-changing behavior that compromises the overall stability and cohesion of a group of autonomous vehicles in an AV network.

**Headway Time [2,18]**: This would prevent the AV from monitoring and regulating AV time gaps. This could allow adversaries to influence headway periods, resulting in dangerous following distances, increased collision risks, and platoon chaos in braking and accelerating.

**Protocol [2,18]**: Message reordering and replay attacks, which target the communication protocol, would render the AV vulnerable. This susceptibility may be exploited by adversaries to disrupt the sequence of messages, which may result in erroneous decision-making, inconsistent state information, and a possible erosion of confidence in the communication infrastructure as a whole.

**Plausibility [2,18]**: The AV will not be able to check data consistency and reasonableness. This could enable adversaries to inject faked or altered sensor readings, resulting in erroneous situational awareness, faulty decision-making, and potentially risky moves inside the AV network.

**Consistency [2,18]**: The AV would lack the ability to effectively cross-validate the information provided by disparate components and sensors. This vulnerability could enable adversaries to exploit inconsistencies or discrepancies in the data, potentially disrupting the coordination and decision-making processes within the AV network.

In our work, the term “anomalies” refers to samples labeled as attacks or abnormal behavior within the context of the VeReMi and Sensor datasets. We acknowledge that these samples may not represent true anomalies in the sense of being entirely novel or generated by unknown mechanisms. Instead, they are known deviations from benign behavior.

Having defined main anomaly types for detecting anomalies in autonomous driving systems, we next show the need for enhancing anomaly detection for such systems.

### 3.2. Anomaly Detection Systems for AVs

The increasing sophistication of anomalies poses a significant threat to VANET systems [5]. Consequently, anomaly detection frameworks play an important role in safeguarding VANET against malicious activities, whether initiated by internal users or external infiltrators [38]. Traditional anomaly detection designs typically rely on single-classifier models which are leveraged to detect the malicious activities in vehicular networks [39]. With recent advancements in AI over the past decade, this design paradigm has paved the way for the development of AI models capable of automatically detecting anomalies in VANET [40]. However, this line of work on using single-classifier models for anomaly detection for AVs has a few shortcomings, as shown next.

### 3.3. Shortcomings of Base Learner Models

While AI models have greatly streamlined the process of anomaly detection, their complexity limits their effectiveness due to the intricate structure of their learning and decision-making processes. The intricate nature of datasets poses a challenge for a single model to properly grasp their subtleties, resulting in difficulty in effectively learning specific subsets and attaining adequate metrics for certain outcomes. This difficulty is widespread among many AI models, such as DT, KNN, SVM, MLP, and others. We emphasize that KNN is a non-parametric method, whereas the rest of the base models used in this work are parametric. Although anomaly detection models have demonstrated high predictive accuracy, there is still room for improvement in achieving better accuracy, precision, recall, and F1-scores, particularly when it comes to errors or attacks. Some AI models suffer from a high false positive rate, as reported in a study on false rates in AI models [41], while others exhibit a high false negative rate, as mentioned in another study [42]. This matter is especially crucial in safety-sensitive applications, such as VANET. As a result, there is an increasing drive to enhance performance and broaden the application of AI models in the field of anomaly detection systems. The need to improve anomaly detection has prompted the use of various ensemble learning techniques [43].

### 3.4. Main Benefits of Popular Ensemble Methods

The selection of appropriate machine learning algorithms for a given application or task is a nuanced process, as each model possesses unique strengths and weaknesses that may be better suited to specific problem domains. This disparity in model performance underscores the importance of carefully evaluating the suitability of individual algorithms based on the characteristics of the problem at hand. For instance, the KNN algorithm relies on the clustering of similar data points around centroids, rendering it sensitive to the number of clusters (K), the presence of class outliers, and the inclusion of irrelevant features. Additionally, KNN can be computationally expensive, particularly for large-scale datasets. In contrast, MLPs often require extensive volumes of training data and may be resource-intensive, while also exhibiting sensitivity to input perturbations. Conversely, Decision Trees are efficient in terms of training time, but can oversimplify problems and lead to overfitting, potentially limiting their generalization capabilities. Given the unique strengths and weaknesses inherent to these machine learning algorithms, the judicious combination of multiple models can be a valuable strategy to improve their overall generalization and performance, particularly in the context of anomaly detection tasks. By leveraging the complementary attributes of diverse algorithms, it is possible to harness their collective strengths while mitigating their individual weaknesses, thus enhancing the robustness and effectiveness of the overall machine learning system which is a must in the safety-critical domain of autonomous driving systems.

Stacking employs a meta-model that is trained using the out-of-fold predictions generated by the base models during cross-validation. The base models, on the other hand, are trained using the entire training dataset and their predictions on the test set are utilized as input features for the meta-model. Stacking may be applied to both regression and classification problems where a meta-model is trained using the numeric predictions or class probabilities obtained from the base models. Contrarily, blending involves utilizing a meta-model that has been trained on the predictions generated by the base models on a separate validation dataset, instead of using predictions made within the same dataset. The training data is divided into two sets: a training set and a validation set. The base models are trained using the training set and their predictions on the validation set are utilized as input features for the meta-model. Blending is less prone to information leaking compared to stacking. However, it requires a smaller amount of training data for the foundation (or base) models.

In our current work, we explore these different types of ensemble learning models in our ensemble learning framework for anomaly detection in autonomous driving systems. We also compare the performances of these methods with just using the base models for anomaly detection tasks for autonomous driving datasets. We perform such comparisons for two different datasets with different characteristics while exploring different evaluation metrics to gain a deeper understanding of our proposed framework.

## 4. Materials and Methods

**Figure 1:**
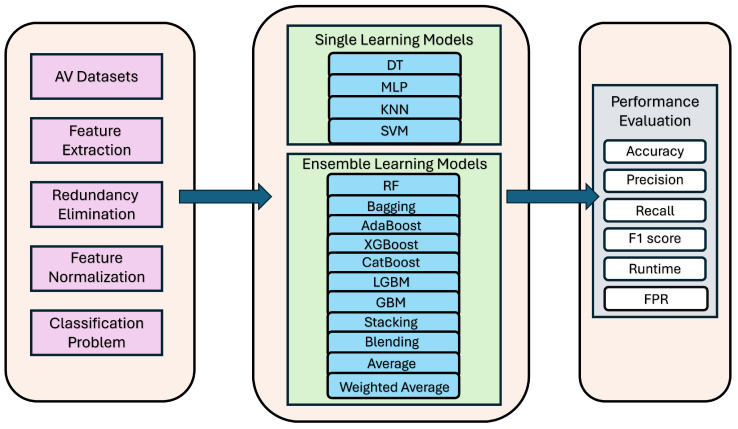
Overview of our ensemble learning framework for anomaly detection in autonomous driving systems. We show preprocessing, main models, and main performance evaluation metrics.

The primary objective of this study is to introduce an ensemble learning pipeline designed to improve performance metrics for anomaly detection in autonomous driving data. The core components of our system are outlined in the overview figure (shown in detail in Figure 1).

### 4.1. End-to-End Framework for Autonomous Driving Systems

**Autonomous Driving Datasets:** For the anomaly categorization of AVs, we utilize two distinct datasets in this study. The VeReMi dataset is a collection of data specifically designed for analyzing the detection of misbehavior in VANETs. This dataset consists of message logs from onboard units (OBUs) and tagged ground truth. It was generated from a simulation environment [17]. Additionally, there is a dataset generated using the Sensor data [44]. This dataset is designed to monitor any abnormal activity detected by an AV using data collected from its ten unique sensors. The Sensor dataset encompasses simulated sensor readings from autonomous vehicles, detecting potential abnormalities across various aspects such as speed, lane positioning, and adherence to communication protocols. We want to emphasize that we followed the data ranges provided by previous studies [18,45] while selecting the Sensor data for each AV.

**Feature Extraction:** Once the datasets are loaded, we extract the main features from each dataset and use them to build our AI models. To defeat adversaries in autonomous driving, feature extraction is critical [46]. Understanding the characteristics that indicate an attack allows you to reduce the overall attack surface. We selected the six most important features for the VeReMi dataset. We want to be clear that we chose the features for the VeReMi dataset based on earlier research [47]. Similarly, we employed all ten features of the Sensor dataset to train our models, based on earlier research [18,45].

**Redundancy Elimination:** In our framework, the crucial step of enhancing dataset integrity involves eliminating redundancy, a prevalent issue particularly in extensive datasets, as it can significantly impair the performance of AI models [48]. In the case of the VeReMi dataset, we tackled redundancy by systematically removing identical rows and those with empty features, thereby streamlining the dataset for more effective analysis. Conversely, in the Sensor dataset, redundancy was not detected. Redundancy elimination is paramount as it ensures that the data fed into AI models is concise, relevant, and free of duplications, ultimately enhancing the accuracy and efficiency of model outcomes.

**Classification Problem:** In our model, we consider both binary and multiclass classification problems. For binary classification, we opted to categorize all types of anomalies within each dataset under the overarching label of ‘anomalous’, effectively splitting our dataset into two classes: ‘benign’ (labeled as ‘0’) and ‘anomalous’ (labeled as ‘1’). This simplification aligns with our objective of anomaly detection regardless of its precise origin for the VeReMi dataset as the Sensor dataset is binary from the beginning. Additionally, this binary classification approach facilitates the implementation of a versatile AI-based framework for anomaly detection in AVs. For the multiclass classification problem, we handled all different anomaly classes for the VeReMi dataset. We emphasize that the details of all classes are provided in Section 5. We emphasize that anomalies, in the strictest sense, refer to input samples that deviate significantly from the training distribution and may be generated by different mechanisms. This aligns with the broader definition used in the anomaly detection literature, where anomalies are often considered rare, unexpected, and difficult to classify due to their diversity and unpredictability [4,49]. The classification task described in this section is designed to distinguish between benign and anomalous samples based on labeled data. This does not constitute anomaly detection in the unsupervised or semi-supervised sense. Rather, it is a supervised classification of known deviations.

**Feature Normalization:** Following basic feature extraction and data balancing, our subsequent step involves feature normalization on the datasets. This phase holds pivotal significance within the data preprocessing pipeline, exerting a notable influence on the efficacy of AI algorithms [50]. The rationale behind normalization lies in mitigating the dominance of any individual feature during analysis. To achieve this, we adopt standard scalar feature scaling, wherein feature values are transformed to attain a mean of 0 and a standard deviation of 1, as outlined in [15]. This approach ensures that each feature contributes proportionately to the overall analysis, thus enhancing the robustness of subsequent AI models.

**Individual Learning Models:** The dataset preprocessing is a crucial step in the machine learning pipeline, followed by a 70/30 split of the data into training and testing sets. This study examines the performance of four well-established AI classification models: DT, MLP, KNN, and SVM. Each of these models requires specific parameter tuning to ensure optimal performance, as detailed in Section 5. The objective of this analysis is to systematically evaluate the strengths and weaknesses of these widely used separate AI classification techniques in the context of the given datasets. They also serve as baselines for our ensemble learning methods.

**Ensemble Learning Models:** In addition to the individual classification models, this study also explores the performance of several ensemble learning techniques, which have gained significant attention for their ability to improve predictive accuracy [51], following the same criteria for preprocessing. The ensemble methods investigated include Bagging, AdaBoost, XGBoost, CatBoost, LGBM, and GBM. Bagging is a technique that creates multiple models from random subsets of the training data and aggregates their predictions to enhance stability and reduce overfitting. AdaBoost, on the other hand, is an adaptive boosting algorithm that iteratively trains weak learners and adjusts their weights based on the performance of previous models, thereby improving the overall model strength. The gradient boosting-based methods, such as XGBoost, CatBoost, LGBM, and GBM, sequentially build Decision Trees, each focusing on the residual errors of the previous model, leading to a more robust and accurate ensemble. Our study also considers the performance of an average ensemble and a weighted average ensemble, which combine the predictions of the individual models. Finally, the analysis explores the potential of Stacking and Blending, which are advanced ensemble techniques that leverage the strengths of multiple base models through a meta-learner. We emphasize that for the latter four ensemble learning classification models, the following four separate models and two ensemble learning models were used as the base/weak learners: DT, RF, MLP, KNN, SVM, and AdaBoost. Each model requires specific parameters to ensure the best possible performance (see Section 5 for full details of parameter tuning).

**AI Model Selection Criteria:** The AI models selected for this study were chosen for two primary reasons. Firstly, their widespread use in existing research on anomaly detection within autonomous driving datasets (e.g., [47,52]) makes them a standard choice. Secondly, by evaluating the classification performance of these AI models on autonomous vehicle datasets, we can effectively compare our methodologies with prior work and maintain consistency with the current literature in AV anomaly detection, particularly when incorporating ensemble learning techniques in our research.

### 4.2. Innovative Value of Our Ensemble Learning Framework in Autonomous Driving Context

We emphasize that the 11 ensemble methods were chosen to represent a wide spectrum of ensemble strategies—bagging, boosting, averaging, and meta-learning—each offering unique strengths for anomaly detection in autonomous systems. This diversity ensures that the system can handle various types of anomalies—ranging from sensor noise to communication faults—by leveraging different learning paradigms. Boosting methods such as XGBoost and CatBoost are particularly effective in handling noisy and imbalanced data, which are common in autonomous driving due to sensor degradation, occlusions, and environmental variability. Bagging methods like Random Forest offer stability and reduce variance, making them suitable for uncertain and dynamic driving conditions. Stacking and Blending allow the integration of heterogeneous base learners, which mirrors the fusion of multi-modal sensor data (e.g., LiDAR, radar, camera). This capability is crucial for autonomous systems that rely on diverse inputs to make real-time decisions. We evaluate all 11 methods across two distinct datasets (VeReMi and Sensor), showcasing their comparative strengths and weaknesses in realistic driving scenarios.

## 5. Foundations of Evaluation

We next show our detailed evaluation. Our evaluation aims to answer the following research questions:

- What are the best base (individual) models for a given autonomous driving dataset?
- Which ensemble method has the best performance for a given autonomous driving dataset?
- What is the performance of different classes of AI models for anomaly detection in our two autonomous driving datasets in terms of accuracy, precision, recall, F1, false positive rate, and runtime?
- What are the limitations and strengths of ensemble learning methods when applied to anomaly detection in autonomous driving systems?

Before showing our detailed evaluation results, we first detail the experimental setup in this section.

### 5.1. Dataset Description

**VeReMi Dataset [17]:** This dataset, known as VeReMi, was introduced as a foundational resource for anomaly detection within the context of autonomous driving, encompassing various types of attacks such as DoS, sybil attacks, and message falsification in VANETs. Each attack scenario within VeReMi is accompanied by real-world data, comprising both readings and ground truth labels, providing a comprehensive basis for algorithmic training and evaluation. The dataset includes AV message logs and an attacker’s ground truth file, facilitating the identification and characterization of attacker behaviors. With a collection of 225 individual scenarios representing diverse attack parameters and five distinct attacker types, VeReMi has been widely recognized as a benchmark dataset in the field of autonomous driving security. Feature selection for VeReMi involved extracting key features that describe AV behavior, specifically focusing on positional coordinates (pos_x, pos_y, pos_z) and velocity components (spd_x, spd_y, spd_z). Irrelevant columns were discarded to streamline the dataset, retaining features deemed essential for anomaly detection tasks. Additionally, a binary classification scheme was applied, whereby all anomalies in the “attackerType” column were generalized as anomalous instances, facilitating the development of anomaly detection models. Moreover, to accommodate multiclass classification, each unique attacker type in the “attackerType” column was designated as a separate class, enabling the development of multiclass anomaly detection models.

**Sensor Dataset [18]:** In our evaluation of the proposed framework, we incorporated the Sensor dataset, adhering closely to the methodology outlined in prior research [18]. This dataset comprises ten essential features, assumed to be inherent to each AV, including formality, location, speed, frequency, correlation, lane alignment, headway time, protocol, plausibility, and consistency sensors. The formality sensor verifies the correctness of message size and header, while the location sensor ensures message delivery to the intended destination. Speed sensor monitors adherence to speed limits, frequency sensor assesses message timing behavior, and correlation sensor evaluates message conformity to predefined specifications. Lane alignment sensor confirms the AV’s position within its lane, headway time sensor maintains distance consistency, and protocol sensor verifies message sequencing. Plausible sensor measures the relative size difference between consecutive messages, and consistency sensor ensures data uniformity across sources. These sensors collectively enable the detection of AV operational modes, distinguishing between normal and malicious behaviors. A delineation of the normal data ranges for the Sensor dataset was provided, facilitating the categorization of anomalous AVs based on deviations from these established norms.

**Summary and Statistics of the Datasets:** Table 2 shows the summary of the two datasets (including dataset size, number of features, number of labels, and attack types). The VeReMi dataset contains both binary and multiclass labels (two and six classes), with six features and a total of 993,834 samples, split into 695,684 for training and 298,150 for testing. It comprises 664,131 normal and 329,703 anomalous samples. In contrast, the Sensor dataset has only binary labels (two classes), 10 features, and 10,000 total samples, divided into 7000 for training and 3000 for testing, with an equal distribution of 5000 normal and 5000 anomalous instances. By dividing the dataset into 70% for training and 30% for testing, we constructed six popular AI classification models (detailed below). Note that we considered both binary and multiclass classification for VeReMi dataset.

### 5.2. Experimental Setup

**Coding Tools:** To make use of several open-source tools and to have our implementation as open-source, we chose the Python 3 programming language along with different AI toolboxes (including Keras and ScikitLearn). We also used other toolboxes (including Pandas).

**AI Models:** We now detail the AI models used in our work.

**(i) Base Learners:** By pairing four popular AI classification algorithms as base learners (which are DT [53], k-KNN [54], SVM [55], and MLP [56]), we evaluate the ensemble learning framework for anomaly detection in AV networks.

**(ii) Ensemble Methods:** The ensemble techniques used in our framework include the CATBoost [57], LGBM [58], ADaBoost [59], XGBoost [60], and GBM [61]. Also, we apply Bagging techniques (i.e., using different instances of the same model), which includes RF [62] and the models mentioned before (i.e., DT, KNN, MLP, LGBM, CAT, SVM, ADA, and RF). In addition, we created a Bagging method [63] training different models (i.e., CATBoost, MLP, SVM, ADABoost, LGBM, RF, GBM, and XGBoost) in parallel instead of different instances of the same model. Moreover, in Stacking, we use all the previous models with the addition of Averaging [64] and Weighted Averaging.

**Hyperparameters:** We provide our main hyperparameter choices for each AI model and each ensemble method used in our work in Appendix A.

Having provided the main experimental setup, we next detail our evaluation results and corresponding findings.

## 6. Evaluation Results

### 6.1. Performance of Ensemble Learning Models

This study aims to evaluate the performance of ensemble learning methods on two distinct datasets, namely VeReMi and Sensor. Furthermore, we conduct a comparative analysis of the ensemble learning models’ performance against their single model counterparts. The primary objective is to ascertain whether the ensemble learning models exhibit superior performance (under different performance metrics) when subjected to identical experimental criteria as the single models.

Ensemble learning techniques amalgamate multiple base learners, such as Decision Trees or neural networks, to yield a collective prediction that often surpasses the individual performance of its constituent models. By leveraging the strengths and mitigating the weaknesses of diverse models, ensemble methods can potentially enhance predictive accuracy, as well as robustness to noise or outliers.

The VeReMi dataset encompasses vehicle remediation data, while the Sensor dataset comprises sensor-based measurements, providing a diverse testbed for evaluating the ensemble learning algorithms. Consistent experimental conditions, including data preprocessing, feature selection, and model hyperparameter tuning, will be maintained across both single and ensemble models to ensure a fair and rigorous comparison in our evaluation experiments.

Through this comprehensive analysis, we aim to elucidate the potential advantages, if any, of employing ensemble learning methods over their single model counterparts in the context of these two datasets. The findings may contribute to a deeper understanding of ensemble learning techniques and their applicability in various domains, ultimately informing decision-making processes and facilitating the development of more robust and accurate predictive models.

#### 6.1.1. Binary Classification

**VeReMi analysis:** For the binary classification analysis of the VeReMi dataset, we took eleven ensemble learning models into consideration, namely the following: RF, Bagging, AdaBoost, XGBoost, CatBoost, LGBM, GBM, Avg, W. Avg, Stacking, and Blending.

Table 3 shows the summary of the evaluation metrics for the classification results for the ensemble methods. As depicted in Table 3, RF, Bagging, Avg, W. Avg, Stacking, and Blending have the highest accuracy of 0.80, indicating that they correctly classify 80% of the instances. Among them, the latter four have the highest precision of 0.83, which means that out of the instances they predicted as positive, 83% were actually positive. However, when considering recall, AdaBoost, XGBoost, CatBoost, and GBM have higher values, with GBM achieving a perfect recall of 1.00. This means that GBM correctly identified all the positive instances, albeit at the cost of lower precision (0.68). In terms of the F1-score, which provides a balanced measure by considering both precision and recall, RF and Bagging have the highest score of 0.86. This indicates that they strike a good balance between precision and recall. The ensemble models Avg, W. Avg, Stacking, and Blending all have the same performance metrics as RF and Bagging, suggesting that they may be aggregating or combining the predictions of these models.

Overall, based on the provided metrics, RF, Bagging, Avg, W. Avg, Stacking, and Blending emerge as the top-performing ensemble models, exhibiting the highest accuracy, precision, and F1-score. Their strong performance can be attributed to their ability to effectively combine multiple Decision Trees, reducing overfitting and improving generalization capabilities.

On the other hand, in the case of single-classifier models as depicted in Table 4, among these single models, DT and KNN have comparable performances, with DT having slightly higher accuracy (0.79) and F1-score (0.84), while KNN has slightly higher recall (0.85). Both DT and KNN exhibit decent precision (0.82 for both) and recall values, suggesting a reasonable balance between accurately identifying positive instances and avoiding false positives. MLP model’s performance is characterized by a high recall but lower precision and accuracy compared to other single models like DT and KNN, which suggests that MLP is adept at identifying positive instances but may struggle with false positives, potentially due to factors like overfitting or the complexity of the problem domain. The SVM model appears to underperform compared to the other single models, with the lowest accuracy (0.55), precision (0.54), recall (0.66), and F1-score (0.60).

**Sensor analysis:** Again, for the binary classification of the Sensor dataset, we used the same eleven models with the same hyperparameters to ensure accurate comparison. CatBoost performed the highest in binary classification for Sensor dataset (Table 5). The other high-performing ensemble models include AdaBoost, XGBoost, and LGBM, all of which have accuracy, precision, recall, and F1-score values of 0.98 or higher. These models also demonstrate exceptional predictive capabilities on the Sensor dataset. It is worth noting that the ensemble models like Stacking and Blending, which aggregate the predictions of multiple base models, also perform very well, with accuracy, precision, and F1-score all above 0.98. The strong performance of CatBoost and the other ensemble models can be attributed to their ability to effectively combine the strengths of multiple base learners, such as Decision Trees or gradient boosting algorithms. By leveraging the diversity of these base models, the ensemble methods are able to capture complex patterns in the data, improve generalization, and mitigate the weaknesses of individual models.

On the other hand, for the single-classifier models on the Sensor dataset, MLP emerges as the top performer, exhibiting the highest accuracy of 0.89, precision of 0.93, recall of 0.93, and an impressive F1-score of 0.93. The MLP’s ability to capture complex patterns and relationships in the data through its multilayered architecture contributes to its superior predictive capabilities. In contrast, the KNN model demonstrates the weakest overall performance, with an accuracy of 0.84 and a relatively lower precision of 0.85, despite its exceptional recall of 0.97. The KNN’s reliance on proximity-based classification, while effective in correctly identifying positive instances, may struggle to achieve a well-balanced trade-off between precision and recall. DT and SVM models exhibit intermediate performance, with DT achieving an accuracy of 0.85 and SVM at 0.88. Both models demonstrate strong precision and recall, resulting in F1-scores in the 0.90–0.92 range. The main insight here is that the absolute gain in accuracy is 11% when using ensemble learning models compared to that of the best single classifier.

**Superiority of Ensemble Methods over Single Methods:** Ensemble models match or outperform the best single models in terms of accuracy, recall, and F1-score, indicating their ability to improve overall performance. For VeReMi dataset, the ensemble models Avg, W. Avg, Stacking, and Blending perform on par with RF and Bagging, likely by combining the predictions of these strong base models in terms of accuracy. Again, Avg, W. Avg, Stacking, and Blending outperform the single classifiers in precision. Moreover, for recall and F1, GBM and RF, Bagging, Avg, W. AVg, Stacking, and Blending models are outperforming the single-classifier models (Table 3 and Table 4). Other single models like MLP, KNN, and SVM generally underperform compared to the ensemble models across multiple metrics. For the Sensor dataset, apart from GBM and Avg, all the ensemble learning models are outperforming single-classifier models in terms of accuracy. Again for precision, recall, and F1-score, most of the ensemble learning models are performing better than the single-classifier models (Table 5 and Table 6). Overall, the ensemble learning models demonstrate competitive or superior performance compared to the single models across key evaluation metrics. This aligns with the general notion that ensemble techniques can leverage the strengths of multiple models and mitigate their individual weaknesses, leading to improved predictive performance and generalization capabilities.

#### 6.1.2. Multiclass Classification

We are exclusively examining the VeReMi dataset for multiclass classification, while our Sensor dataset only consists of two classes. Next, we will analyze and compare the assessment metrics of ensemble models and single models to assess their performance.

From Table 7, we see the Stacking and Blending ensemble models emerged as the top performers, achieving the highest accuracy of 0.72. These advanced ensemble techniques outperformed the other ensemble models. Additionally, the stacking model demonstrated the strongest precision at 0.79, closely followed by the blending model at 0.78. The RF ensemble model also exhibited relatively high precision at 0.79. The XGBoost, CatBoost, and LGBM ensemble models achieved a perfect recall of 1.00, indicating their ability to correctly identify all positive instances. The Stacking and Blending ensemble models also demonstrated high recall, with a value of 0.96. Aligning with their superior accuracy and balanced performance, the Stacking and Blending ensemble models attained the highest F1-scores at 0.87. This showcases their ability to strike an optimal trade-off between precision and recall. The RF ensemble model also demonstrated a strong F1-score of 0.86. The superior performance of the stacked and blended ensemble models can be attributed to their capacity to effectively integrate the predictions of multiple base models. These advanced ensemble techniques can capture more complex relationships and patterns in the data, leveraging the strengths of individual models while mitigating their weaknesses. In contrast, while the XGBoost, CatBoost, and LGBM ensemble models achieved near-perfect recall, their precision and overall F1-scores were lower compared to the Stacking and Blending approaches. This suggests that the Stacking and Blending ensembles have a more balanced trade-off between precision and recall, resulting in better predictive performance.

Again, we evaluate the performance of single-classifier models on the multiclass classification of the VeReMi dataset. Table 8 shows the performance of single-classifier models on multiclass classification. Based on the metrics in the table, the KNN model exhibits the strongest overall performance. KNN has an accuracy of 0.65, precision of 0.77, recall of 0.95, and an F1-score of 0.85. KNN’s high recall of 0.95 indicates its proficiency in correctly identifying positive instances, while its balanced precision and F1-score suggest a well-rounded predictive capability. DT model follows closely, with an accuracy of 0.66, precision of 0.72, recall of 0.97, and an F1-score of 0.83. DT’s high recall of 0.97 showcases its ability to capture the majority of positive instances, though its precision is relatively lower compared to KNN. The MLP and SVM models exhibit the best performance in terms of accuracy, achieving 67%. While the MLP and SVM achieve a perfect recall of 1.00, their lower precision and F1-scores of 0.80 suggest they struggle to maintain a balance between precision and recall.

Therefore, we conclude that for multiclass classification on VeReMi dataset ensemble learning models perform with 5% higher accuracy than single-classifier models.

#### 6.1.3. Runtime Analysis

Subsequently, we assess the execution time of both ensemble and single-classifier models on our two datasets on the same testing size. We recorded the training and prediction times for each of the classifier models. We perform this analysis to evaluate the efficacy of both individual models and ensemble models.

**Figure 2:**
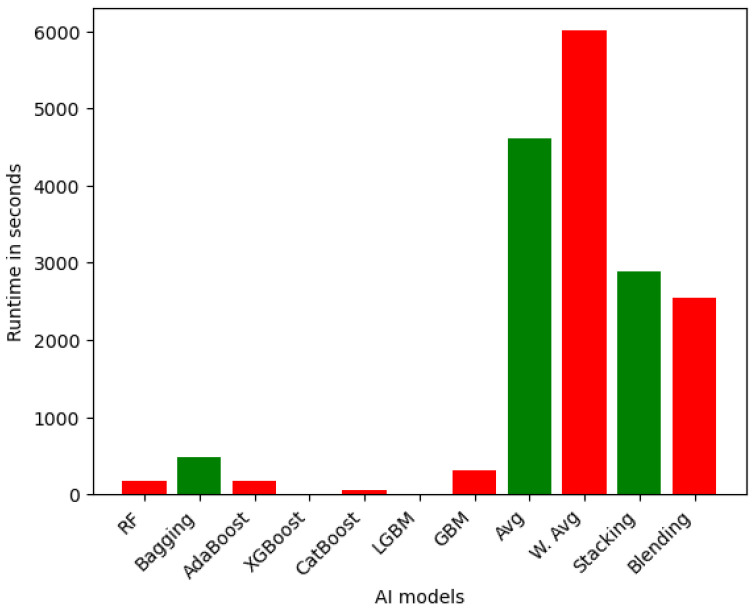
Runtime of ensemble learning classifier models for binary classification on the VeReMi dataset. LGBM is the most efficient ensemble classifier model in binary classification for the VeReMi dataset.

**Efficiency of Ensemble Models on VeReMi dataset:** First, we capture the time to generate the outcome of each ensemble model for VeReMi (binary class). Figure 2 depicts the runtime for ensemble learning models for the VeReMi binary dataset. The LGBM and XGBoost ensemble models demonstrate the highest computational efficiency, with runtimes of 4.00 s and 2.50 s, respectively. These gradient boosting-based models are able to achieve fast training and prediction times, leveraging optimized algorithms and parallel processing capabilities. In contrast, other ensemble models exhibit a wider range of runtimes, with some, like Random Forest (181.15 s) and CatBoost (62.20 s), being relatively efficient, while others, such as Bagging (481.38 s), GBM (316.75 s), and the more complex Stacked (2886.90 s) and Blended (2538.86 s) models, require significantly longer computational times. This is due to the additional overhead associated with training and aggregating the predictions of multiple base learners. At the extreme end, the W. Avg and simple Avg ensemble techniques demonstrate the slowest runtimes, taking over 1.5 h and over 1 h, respectively, reflecting the substantial computational burden of combining six of the models.

**Figure 3:**
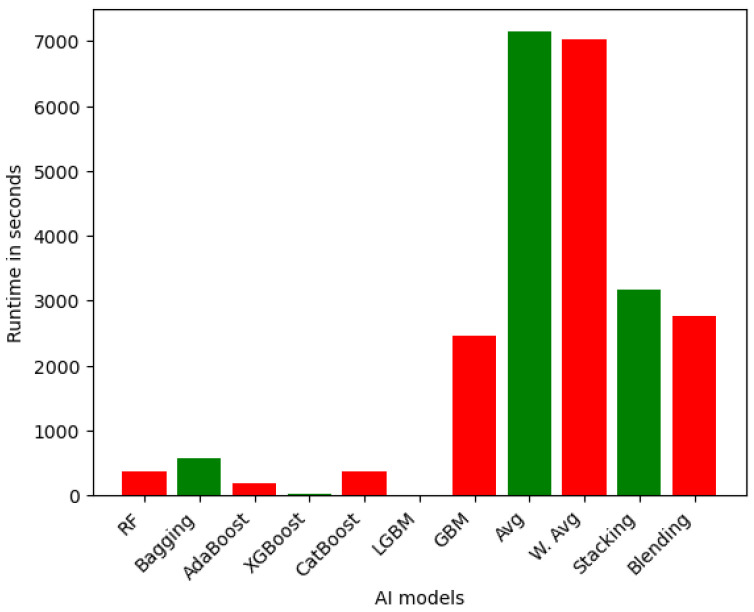
Runtime of ensemble learning classifier models for multiclass classification on the VeReMi dataset. LGBM is the most efficient ensemble model to generate results in multiclass classification.

However, for the case of multiclass classification, the performance of ensemble learning models was slightly higher for all the models. Figure 3 depicts the runtime for ensemble learning models for multiclass classification. Among the ensemble models, again the LGBM emerges as the most efficient, requiring only 9.18 s to complete its computations. This can be attributed to the lightweight and optimized nature of the LGBM algorithm, which leverages efficient tree-building techniques and parallel processing capabilities to achieve fast training and prediction times. In contrast, the GBM ensemble exhibits a long runtime at 2456.24 s, highlighting the substantial computational burden associated with more complex gradient boosting-based approaches. The RF (369.01 s) and CatBoost (366.36 s) ensemble models demonstrate runtimes that are generally faster than the more sophisticated ensemble techniques like Stacking (3177.28 s) and Blending (2771.32 s). This is due to the additional overhead required to train and combine the predictions of multiple base models in the advanced ensemble methods. The Avg and W. Avg ensemble models show the longest runtimes, taking over 7000 s (approximately 2 h) to complete, similar to their performance for the binary class.

**Figure 4:**
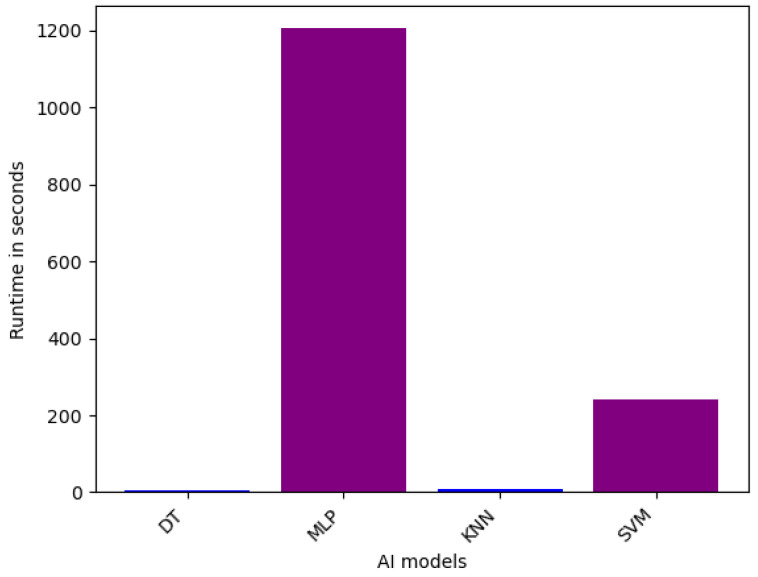
Runtime of single classifiers for binary classification on the VeReMi dataset. DT is the most efficient single-classifier model for VeReMi.

**Efficiency of Single Models on VeReMi dataset:** For the single models for binary classification, the runtime performance is shown in Figure 4. From the figure, it is evident that the MLP model exhibits the longest runtime, requiring 1204.27 s to complete its computations. This can be attributed to the inherent complexity and extensive parameter tuning required for training Multilayer Perceptron architectures, which often involve a substantial number of layers and interconnections. In contrast, the DT model demonstrates the fastest runtime at 6.28 s, highlighting the computational efficiency of Decision Tree-based algorithms, which rely on recursive partitioning of the feature space. The KNN and SVM models fall between these extremes, with runtimes of 10.02 s and 240.87 s, respectively. The relatively longer runtime of SVM compared to KNN can be explained by higher computational complexity associated with kernel function’s optimization.

**Figure 5:**
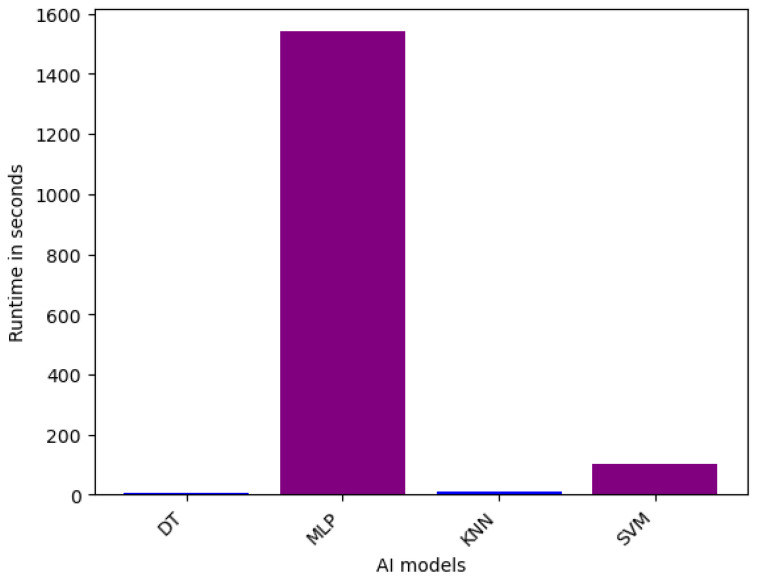
Runtime of single-classifier models for multiclass classification on the VeReMi dataset. Again, DT is the most efficient single-classifier model.

For multiclass classification on single models, Figure 5 provides the runtime in seconds for several single-classifier models, including DT, MLP, KNN, and SVM. Among the single models, the DT demonstrates the fastest runtime, requiring only 5.94 s to complete its computations. This efficiency can be attributed to the inherent simplicity and low computational complexity of Decision Tree-based algorithms, which rely on iterative partitioning of the feature space. In contrast, the MLP model exhibits the longest runtime of 1540.30 s, reflecting the substantial computational overhead associated with training and tuning Multilayer Perceptron architectures, which often involve a large number of parameters and layers. The KNN and SVM models fall between these extremes, with runtimes of 11.05 s and 103.93 s, respectively. The longer runtime of SVM compared to KNN can be explained by the higher computational complexity involved in optimizing the kernel function and solving the quadratic programming problem during the training phase.

**Figure 6:**
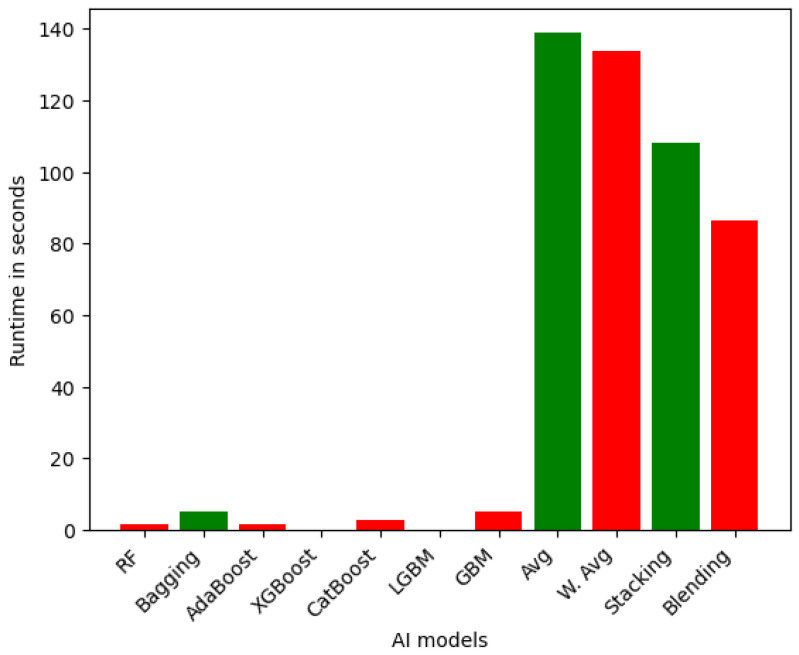
Runtime of ensemble classifier models on the Sensor dataset. LGBM is the most efficient ensemble learning classifier model for the Sensor dataset.

**Efficiency of Ensemble Models on Sensor dataset:** Now, we compute the runtime for ensemble models on our Sensor dataset. Figure 6 presents the runtime in seconds for various ensemble learning models. The results showcase a wide range of computational efficiency across the different ensemble techniques. At the top of the efficiency spectrum, we find the LGBM and XGBoost models, which require only 0.087 s and 0.109 s, respectively, to complete their computations. These gradient boosting-based ensemble methods have been specifically optimized for computational efficiency, leveraging techniques such as parallel processing and memory-efficient data structures. The ensemble models, including Random Forest (1.50 s), AdaBoost (1.50 s), and CatBoost (2.69 s), also demonstrate relatively fast runtimes, outperforming the more complex ensemble approaches like Bagging (5.34 s), GBM (5.35 s), and the Stacking (108.23 s) and Blending (86.26 s) models. At the lower end of the efficiency scale, the Averaging (138.83 s) and Weighted Averaging (133.91 s) ensemble models exhibit the longest runtimes, reflecting the computational burden associated with aggregating the predictions of a large number of base models.

**Figure 7:**
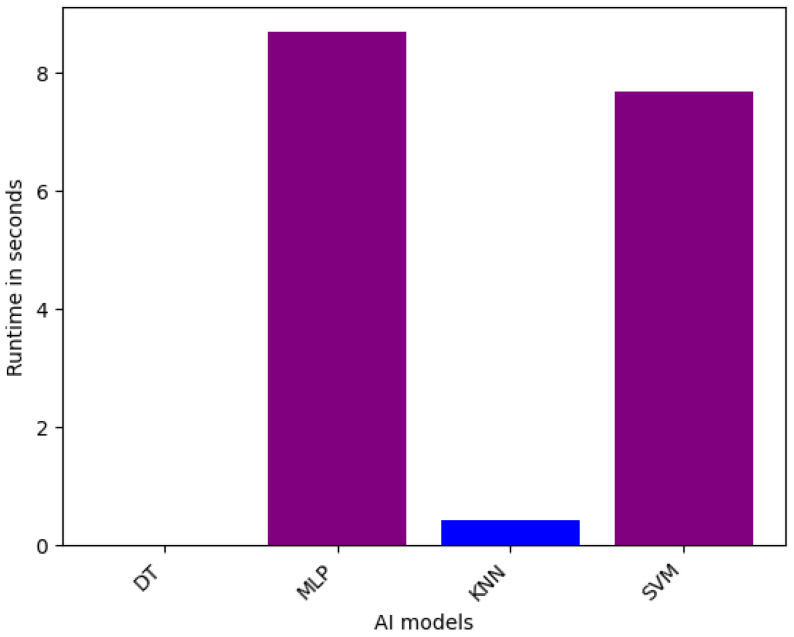
Runtime of single-classifier models on the Sensor dataset. We see that DT is the most efficient single-classifier model for the Sensor dataset.

Again for the runtime calculation on single models, Figure 7 shows the runtime performance. As depicted in the table, DT is the quickest model to generate the outcome for the Sensor dataset. KNN holds the second position, having a runtime of 0.43 s. SVM and MLP take 7.68 and 8.69 s, respectively, to generate results, indicating MLP as the slowest among these four models. The reason behind this is that the multiple layers of neural network make the computation a bit more complex.

In summary, we provided detailed runtime comparisons across all models (Figure 2, Figure 3, Figure 4, Figure 5, Figure 6 and Figure 7), showing that while Stacking and Blending are among the most accurate, they are also the most computationally intensive. In contrast, models like LGBM and XGBoost offer a favorable balance between performance and efficiency, completing tasks in under one minute. For single models, DT and KNN have the best efficiency.

#### 6.1.4. False Positive Rate Analysis

We now conduct the false positive rate analysis for both the VeReMi binary and multiclass tasks, as well as for the Sensor dataset, across the ensemble learning models and the single models. The objective is to determine which models have a higher false positive rate (i.e., which models incorrectly classify a sample as benign or anomalous, or the reverse).

**Figure 8:**
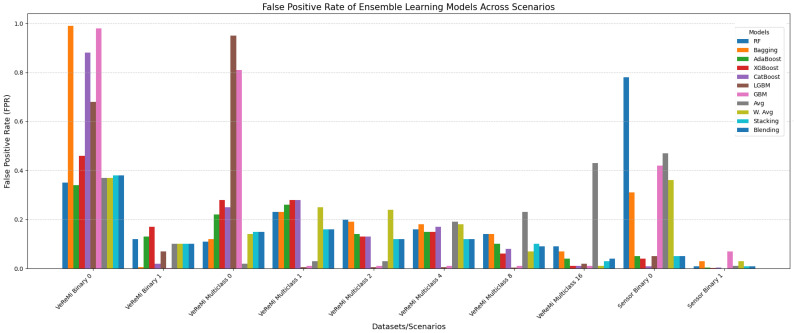
False positive rate of 11 ensemble learning models for VeReMi and Sensor datasets.

Figure 8 compares the false positive rates (FPRs) of 11 ensemble learning models across the VeReMi dataset (binary and multiclass) and the Sensor dataset (binary). A high FPR indicates that the model incorrectly classifies a large number of negative instances as positive, which is undesirable as it reflects poor model performance. Conversely, a low FPR indicates that the model makes fewer false positive errors, signifying better performance. RF, AdaBoost, and Stacking consistently demonstrate low FPRs, indicating robust performance across both datasets. Bagging, CatBoost, LGBM, and GBM show variable performance, with some models exhibiting high FPRs for certain tasks. For instance, Bagging has a notably high FPR of 0.99 in the binary classification of VeReMi, indicating a high rate of incorrect positive classifications. A notable trend is that while some models perform exceptionally well in binary classification, their performance drops in multiclass scenarios. The performance of XGBoost and CatBoost varies significantly, highlighting the importance of model selection based on the specific application. In summary, RF, AdaBoost, and Stacking are generally the top performers, while models like Bagging and GBM need careful consideration due to their higher false positive rates in certain cases.

**Figure 9:**
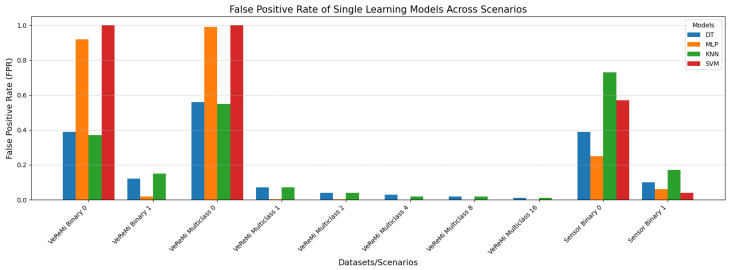
False positive rate of 4 single learning models for VeReMi and Sensor datasets.

On the other hand, Figure 9 presents the false positive rates (FPRs) of four single learning models (DT, MLP, KNN, and SVM) for the VeReMi dataset (binary and multiclass) and the Sensor dataset (binary). DT and KNN exhibit relatively lower FPRs for multiclass VeReMi with increasing values, indicating better performance in more complex scenarios. MLP shows a high FPR of 0.99 for binary classification but drastically improves for multiclass with near-zero FPRs, suggesting high variability based on task complexity. SVM has a perfect FPR of 1 for binary classification in the VeReMi dataset, signifying poor performance, but performs better with an FPR of 0 for multiclass. In the Sensor dataset, DT and KNN show moderate FPRs, whereas MLP and SVM exhibit lower FPRs, indicating better performance. Overall, DT and KNN are more consistent across tasks, while SVM and MLP demonstrate significant variability depending on the dataset and classification type.

Having shown the main performance metrics for both datasets, we next show the effect of hyperparameter tuning on the performances of different single and ensemble models in detecting anomalies.

### 6.2. Effect of Hyperparameters of Ensemble Learning Models

#### 6.2.1. VeReMi Dataset

Examining various hyperparameter settings in ensemble learning methods reveals subtle effects on model performance indicators for the VeReMi dataset (Table 9).

Regarding the RF classifier, raising the maximum depth (max_depth) parameter from 4 to 50 led to improvements in accuracy, precision, and F1-score, but there was a decrease in recall. Similarly, the Bagging ensemble showed similar patterns, with an improvement in accuracy, precision, and F1-score when the maximum depth parameter was increased, except for recall. In a similar manner, the AdaBoost model showed a slight enhancement in accuracy, precision, and F1-score when the maximum depth was raised from 4 to 6, while still maintaining the model’s strong recall performance. The XGBoost classifier had a clear trend, where increasing the maximum depth parameter from 4 to 6 resulted in improved accuracy, precision, and F1-score, but a drop in recall. Upon exploring the CatBoost model, fine-tuning the learning rate and tree depth parameters resulted in a marginal improvement across all assessment measures except for recall. In contrast, the LGBM model demonstrated enhanced accuracy, precision, and F1-score as the learning rate was raised from 0.5 to 0.8 and the number of boosting rounds was increased from 100 to 500. However, there was a modest decrease in recall. The GBM showed significant improvements in accuracy, precision, and F1-score when the learning rate was increased from 0.01 to 0.5 and the maximum depth was increased from 10 to 50. However, the recall decreased when the learning rate and maximum depth were increased.

Overall, the analysis suggests an interesting trade-off in the performance of bagging and boosting models with respect to the max_depth hyperparameter. Specifically, increasing the max_depth parameter does not necessarily lead to a uniform enhancement in model performance across all evaluation metrics. While higher max_depth values may yield improvements in accuracy, precision, and F1-score, the results indicate that the maximum recall can be obtained with a lower max_depth of 4. Elevating the learning rate parameter (‘lr’) manifests in heightened accuracy across CatBoost, LGBM, and GBM models. Furthermore, our observations indicate that augmenting the number of estimators did not yield discernible enhancements across any of the models under consideration. In terms of accuracy, RF, Bagging, AdaBoost, and GBM are performing the best. AdaBoost is the best performer in the case of precision. For recall, RF, Bagging, and GBM depict the perfect score. Lastly, for F1-score, RF and Bagging perform the best.

#### 6.2.2. Sensor Dataset

In the realm of ensemble learning, hyperparameter tuning plays a pivotal role in optimizing the performance of various models. Table 10 presents a comparative analysis of different ensemble learning models for the Sensor dataset, namely RF, Bagging, AdaBoost, XGBoost, CatBoost, LGBM, and GBM, with respect to their hyperparameters and performance metrics. The hyperparameters under consideration are ‘max_depth’ and ‘n_estimators’ for RF, Bagging, AdaBoost, and XGBoost, while ‘lr’ (learning rate), ‘depth’, ‘num_boost_round’, and ‘max_depth’ are considered for CatBoost, LGBM, and GBM. The performance metrics used for evaluation are accuracy (Acc), precision (Prec), recall (Rec), and F1-score. Upon analyzing the table, it is evident that the tuning of hyperparameters significantly influences the performance of these models. For instance, in RF, Bagging, and AdaBoost, an increase in ‘max_depth’ and ‘n_estimators’ generally leads to an improvement in all performance metrics, although this trend is not absolute. In the case of XGBoost, the model achieves the highest scores in all metrics at a ‘max_depth’ of 4 and ‘n_estimators’ of 100, indicating that the model’s performance does not always improve with an increase in these hyperparameters. For CatBoost, LGBM, and GBM, the tuning of ‘lr’, ‘depth’, ‘num_boost_round’, and ‘max_depth’ also yields varying results. Notably, CatBoost achieves perfect scores in most metrics at an ‘lr’ of 0.03, ‘depth’ of 6, and ‘num_boost_round’ of 10.

Overall, for bagging classifiers, RF shows a slight improvement in performance when max_depth is increased. However, keeping the max_depth at 50 and changing the n_estimators results in significant change in performance. In the case of boosting classifiers, a lower max_depth produces high performance for the AI models. Generally, increasing the n_estimators will increase the accuracy slightly for AdaBoost and XGBoost. A learning rate of 0.5 seems to produce the highest performance as well for CatBoost, LGBM, and GBM.

#### 6.2.3. Guidelines and Insights from Hyperparameter Tuning

We now include practical heuristics and tuning strategies based on our experimental findings from the above experiments.

**(A) General Tuning Guidelines:** For tree-based models (e.g., Random Forest, XGBoost, LGBM), we recommend starting with a moderate max_depth (e.g., 6–10) and gradually increasing it while monitoring overfitting. For boosting models, a smaller learning_rate (e.g., 0.01–0.1) combined with a higher number of estimators (e.g., 100–500) often yields better generalization. For Stacking and Blending, we suggest using diverse base learners (e.g., DT, MLP, SVM) and a simple meta-learner (e.g., logistic regression or shallow Decision Tree) to balance performance and runtime.

**(B) Dataset-Specific Insights:** On the Sensor dataset, shallow models with fewer estimators performed well due to the dataset’s smaller size and lower complexity. On the VeReMi dataset, deeper trees and more estimators improved performance, but at the cost of increased runtime—highlighting the importance of balancing depth and efficiency.

**(C) Runtime-Aware Tuning:** For real-time applications, we recommend prioritizing models like LGBM and XGBoost, which showed strong performance with minimal runtime. For offline or batch processing, more complex ensembles like Stacking and Blending can be used with higher n_estimators and deeper base models.

**(D) Hyperparameter Search Strategy:** We suggest using random search or Bayesian optimization over grid search for efficiency, especially when tuning multiple models in ensemble configurations.

### 6.3. Comparative Analysis and Summary of Evaluation Results

In this subsection, we present a comprehensive summary of the performance of various single models and ensemble methods across two prominent autonomous driving datasets: Sensor and VeReMi. Our summary, provided in Table 11, focuses on three main aspects: accuracy-related metrics, false positive rate, and runtime efficiency.

**Performance Metrics (Accuracy, Precision, Recall, F1):** Across both datasets, ensemble methods consistently outperformed individual models in terms of accuracy, precision, recall, and F1-score. For the Sensor dataset, the best-performing models included CatBoost, LGBM, Stacking, Blending, and AdaBoost. In the VeReMi dataset, top models included RF, Bagging, Averaging, Weighted Averaging, Stacking, Blending, LGBM, and DT. When combining results from both datasets, the models that performed best overall were Blending, Stacking, CatBoost, LGBM, RF, AdaBoost, and DT, highlighting the strength and versatility of ensemble learning in varied anomaly detection scenarios.

**False Positive Rate (FPR):** For minimizing false alarms, ensemble methods again proved effective. On the Sensor dataset, models such as RF, AdaBoost, Stacking, CatBoost, Bagging, LGBM, and MLP achieved the lowest FPR. For the VeReMi dataset, both ensemble and single models such as RF, AdaBoost, Stacking, DT, and KNN were most effective. Overall, the most reliable models for low FPR were RF, AdaBoost, and Stacking, emphasizing their suitability for precision-critical applications like real-time anomaly detection tasks for autonomous driving systems.

**Runtime Efficiency:** Runtime analysis was conducted to assess model suitability for real-time or resource-constrained environments. The fastest models, which completed their tasks in under one minute across all variants, included RF, Bagging, AdaBoost, XGBoost, CatBoost, LGBM, GBM, DT, MLP, KNN, and SVM. These models are ideal for rapid deployment on AV systems. Models with moderate runtime (between one and ten minutes) included Averaging, Weighted Averaging, Stacking, and Blending on the Sensor dataset, and RF, CatBoost, Bagging, GBM, and SVM on VeReMi. These are suitable for fog computing environments where moderate computational power is available. The slowest models, taking over ten minutes to run, were primarily ensemble approaches like Stacking, Blending, Averaging, Weighted Averaging, and also MLP on the VeReMi dataset. While accurate, these models may require additional optimization or work for offline analysis.

**Overall Best Models:** Based on a combined assessment of classification performance (accuracy, precision, recall, F1-score), false positive rate, and runtime efficiency, the top-performing models are AdaBoost, LGBM, RF, CatBoost, and Stacking. These models consistently demonstrate high predictive accuracy, low false alarms, and fast or reasonable execution times, making them well-suited for deployment in real-time autonomous driving environments.

Having presented our detailed evaluation of our ensemble framework, we next present the main discussion and limitations.

### 6.4. Interperatability Analysis of Top Features for Different Methods

We now provide analysis for how the decision-making is made for different single and ensemble methods, focusing on feature importance analysis. In the interest of space, we will only present full analysis for the Sensor dataset since it has more features.

**Figure 10:**
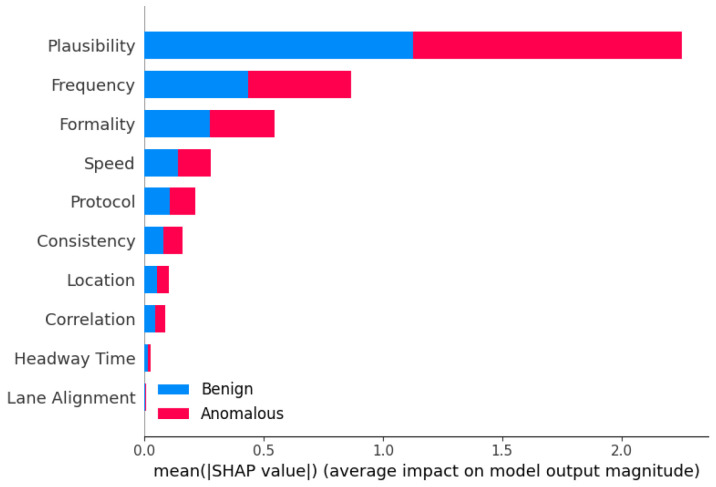
Feature importance of MLP classifier.

**Figure 11:**
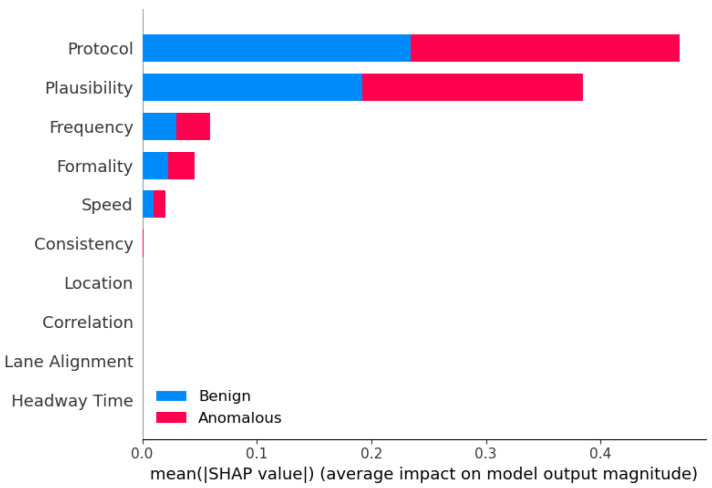
Feature importance of KNN classifier.

**Figure 12:**
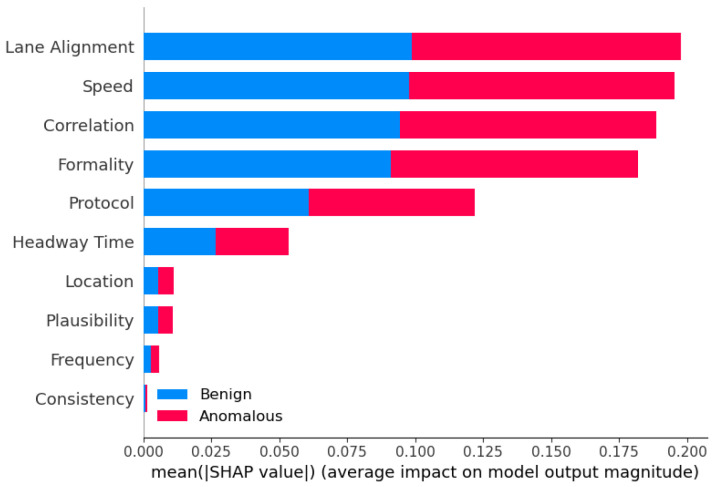
Feature importance of SVM classifier.

**Figure 13:**
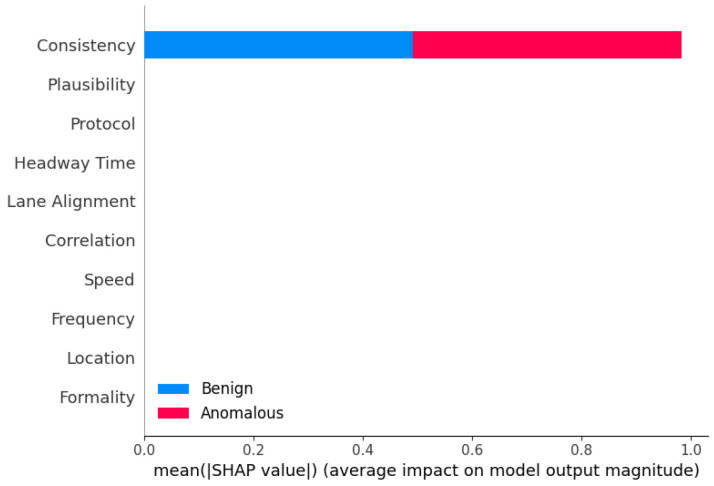
Feature importance of DT classifier.

**Single-AI Models: **Figure 10, Figure 11, Figure 12 and Figure 13 show the feature importance for the single-AI classifiers for Sensor dataset. These figures present SHAP-based global feature importance for four classifiers—MLP, KNN, SVM, and Decision Tree—applied to the Sensor dataset. Each subplot ranks the input features by their contribution to the model’s predictions, highlighting which variables most strongly influence classification outcomes. While the exact ranking differs across models, certain features consistently exhibit high importance across multiple classifiers (such as plausibility, protocol, consistency, and lane alignment), indicating their robust predictive value, whereas others show model-specific relevance. This comparison underscores how different algorithms prioritize features differently, reflecting variations in their underlying decision-making processes.

**Figure 14:**
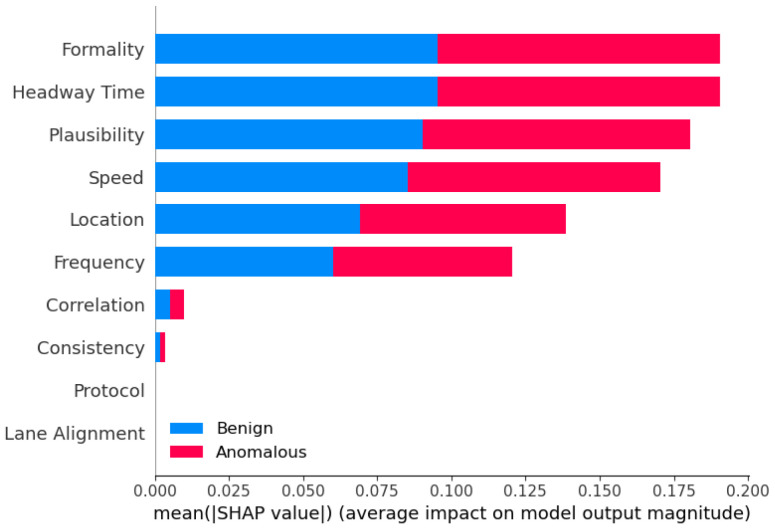
Feature importance of RF classifier.

**Figure 15:**
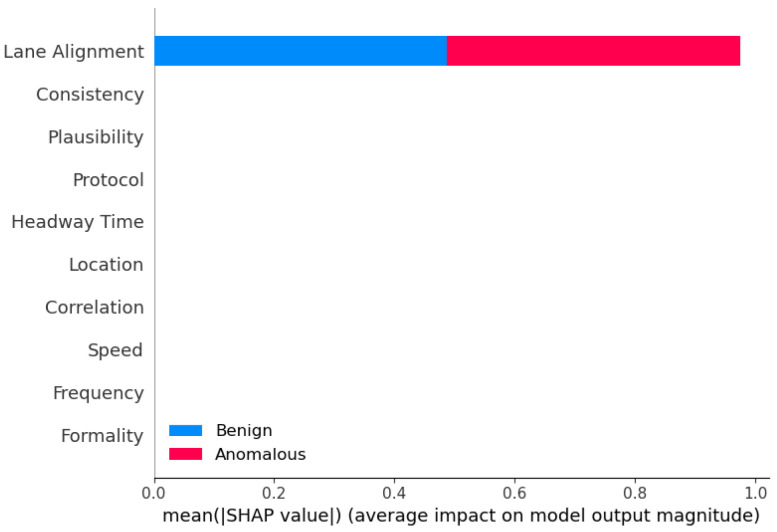
Feature importance of AdaBoost.

**Figure 16:**
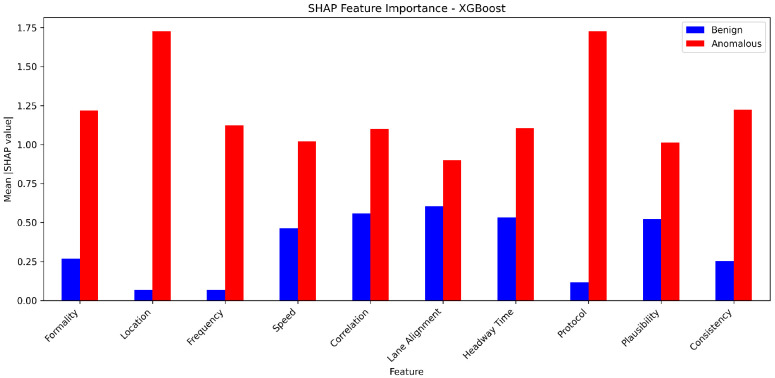
Feature importance of XGBoost.

**Figure 17:**
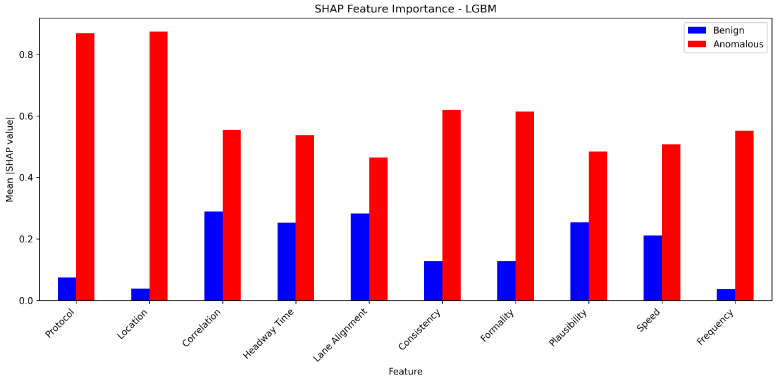
Feature importance of CatBoost.

**Tree-based Ensemble Methods:**Figure 14, Figure 15, Figure 16 and Figure 17 show the importance of global SHAP-based features for four ensemble classifiers based on trees—Random Forest (RF), AdaBoost, XGBoost and CatBoost—evaluated on the Sensor dataset. In each subplot, the most influential features for each model are highlighted, with certain characteristics regularly appearing as the top contributors in all classifiers. For instance, features such as Formality, Lane Alignment, and Protocol stand out as most significant in their respective models. This analysis reveals both consensus and diversity among ensemble methods regarding which input variables most impact prediction, with some features demonstrating prominent importance across multiple models.

**Figure 18:**
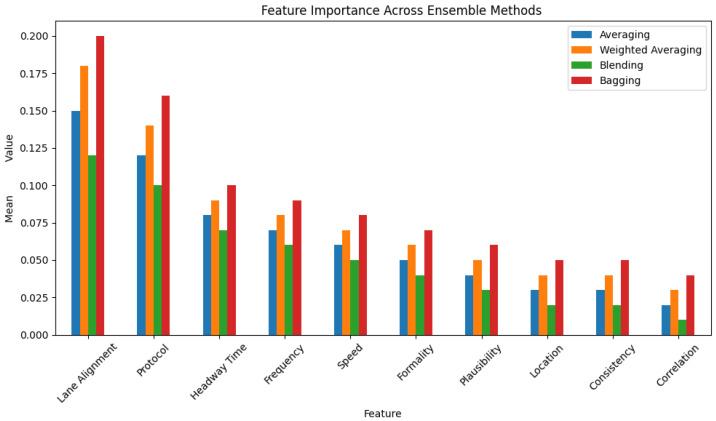
Feature importance for non-tree-based ensemble learners for Sensor dataset.

**Non-Tree-based Ensembles:** For those classifiers, we cannot use SHAP for feature importance. Thus, we leveraged sklearn for feature importance analysis for these classifiers. Figure 18 shows a summary of the comparison of the importance of characteristics across four ensemble methods: Averaging, Weighted Averaging, Blending, and Bagging, visualized below. Lane Alignment and Protocol consistently rank as the most influential features across all ensemble methods. Bagging assigns the highest importance to Lane Alignment and Protocol, indicating strong reliance on these features. Weighted Averaging also emphasizes Lane Alignment and Protocol, but distributes importance more evenly across other features. Blending and Averaging show lower overall importance values, suggesting less feature discrimination. Features such as correlation, consistency, and location have relatively low importance across all methods.

## 7. Discussion

### 7.1. Main Insights and Related Discussion

#### 7.1.1. Superiority of Ensemble Learning over Single Models

The evaluation results clearly demonstrated the superior performance of ensemble learning models compared to single models for the anomaly detection task in autonomous driving datasets. Across both the VeReMi and Sensor datasets, ensemble techniques such as Random Forest, Bagging, AdaBoost, XGBoost, CatBoost, and LGBM consistently outperformed single models including Decision Trees, Multilayer Perceptrons, and Support Vector Machines in terms of accuracy, precision, recall, and F1-score metrics. This can be attributed to the fundamental principle of ensemble learning, which involves combining multiple diverse models to leverage their collective strengths while mitigating individual weaknesses. By amalgamating predictions from various base learners, ensemble methods can effectively capture complex patterns and nuances within the data, resulting in improved predictive capabilities and robustness against outliers.

By evaluating different ensemble methods across both simulated VANET (VeReMi) and sensor-based (Sensor) datasets, we demonstrate their adaptability to different types of autonomous driving environments—ranging from communication-based anomalies to physical sensor deviations. The innovative value lies not only in the individual models but in their synergistic combination. Our results show that certain ensembles outperform individual models significantly, especially in detecting subtle anomalies that may be missed by standalone (or individual) classifiers.

#### 7.1.2. Trade-Off Between Performance Metrics and Hyperparameter Tuning

The analysis of hyperparameter effects on model performance revealed intriguing trade-offs between different evaluation metrics. Notably, increasing the maximum depth parameter (max_depth) for ensemble models often led to improvements in accuracy, precision, and F1-score. However, this came at the cost of a decrease in recall, indicating a trade-off between capturing positive instances and minimizing false positives. This finding highlights the importance of careful hyperparameter tuning and the need to strike a balance between different performance goals, depending on the specific requirements of the anomaly detection task. For instance, in safety-critical applications like autonomous driving, a higher emphasis may be placed on maximizing recall to ensure that potential anomalies are not missed, even if it results in a higher false positive rate.

#### 7.1.3. Computational Efficiency Considerations and Optimizations for Deployment

While ensemble learning models demonstrated superior predictive performance, the analysis of runtime efficiency revealed a significant computational overhead associated with certain ensemble techniques, particularly the more complex approaches such as Stacking and Blending. Models like LGBM and XGBoost exhibited remarkable computational efficiency, owing to their optimized algorithms and parallelization capabilities. This observation underscores the importance of considering the trade-off between model performance and computational complexity, especially in real-time or resource-constrained environments. In scenarios where computational resources are limited, or low latency is critical, the choice of ensemble method should be carefully evaluated, potentially favoring more computationally efficient techniques (e.g., LGBM or XGBoost), over the more complex but accurate models (e.g., Stacking or Blending).

We acknowledge the need for optimizing ensemble models for real-time deployment. Potential strategies include the following: (i) model pruning and compression to reduce complexity, (ii) parallelization and hardware acceleration (e.g., GPU-based inference), and (iii) selective ensemble deployment, where simpler models are used for real-time inference and complex ensembles are reserved for offline analysis or periodic retraining. We suggest that advanced ensemble methods may be more suitable for fog or edge computing environments, where moderate computational resources are available, while lightweight models like LGBM and XGBoost are better suited for onboard real-time inference. We plan to explore adaptive and online learning strategies that allow ensemble models to update incrementally, reducing retraining costs and improving responsiveness in dynamic environments.

### 7.2. Use of AI Notation Instead of ML

We emphasize that the use of the term AI for the models involves a slight abuse of the standard notation of ML. While we agree that the term “AI” is broad and sometimes inconsistently applied, it is widely accepted in the academic literature to refer to a range of ML algorithms—including DT, KNN, SVM, and MLP—as part of the broader field of Artificial Intelligence. For instance, a recent comparative study explicitly includes DT, KNN, and SVM under the umbrella of AI-driven supervised classification models [65]. Similarly, the work [66] investigates the role of DT, KNN, SVM, and MLP in classifying diabetic patient datasets. It is published in the Artificial Intelligence: Theory and Applications conference proceedings, further validating the inclusion of these models within AI research. Therefore, our use of the term “AI models” aligns with standard academic conventions.

### 7.3. Usage of MLP and Traditional AI Models

We note that deeper architectures can offer improved performance in certain contexts. However, our choice of an MLP with two hidden layers as a base model was guided by the complexity of the dataset and the need to balance model performance with computational efficiency. As noted in the literature, MLPs with two or more hidden layers are capable of modeling complex nonlinear relationships and are considered part of the deep learning family when they include multiple hidden layers [67]. While deep learning models have shown superior performance in many domains, traditional ML models remain widely used, especially when data is limited (such as the Sensor dataset case). Studies have shown that in such scenarios, models like SVM, DT, and KNN can perform comparably or even better than deep neural networks [68]. Our selection of models was thus motivated by practical considerations and the characteristics of the dataset.

### 7.4. Limitations

#### 7.4.1. Limited Dataset Diversity

In our paper, we primarily give a complete treatment of our ensemble learning framework for two well-known datasets, with the main focus on the binary-class anomaly detection classification. These two datasets cover specific security scenarios for autonomous driving systems. The VeReMi dataset contains vehicle telemetry data, including various VANET attack types like denial of service, Sybil attacks, and message falsification, as detailed by [69]. In contrast, the Sensor dataset provides simulated sensor measurements from autonomous vehicles, designed to capture anomalies across multiple dimensions such as speed, lane positioning, and communication protocol adherence.

While the study utilizes two real-world datasets, namely the VeReMi dataset and the Sensor dataset, these datasets may not fully capture the diverse range of scenarios and anomalies that could occur in real-world autonomous driving environments. The VeReMi dataset has primarily focused on simulated vehicular ad hoc networks, while the Sensor dataset is a simulated dataset based on predefined sensor measurements. Real-world autonomous driving systems operate in highly complex and dynamic environments, and the anomalies they encounter may exhibit characteristics that are not adequately represented in these datasets. Consequently, the performance of the proposed ensemble learning framework on these datasets may not be generalized seamlessly to other real-world scenarios, potentially limiting its applicability in practical deployments. However, we emphasize that VeReMi and Sensor are widely used benchmark datasets in the autonomous driving and VANET anomaly detection literature [70,71,72]. Their use allows for reproducibility and comparative analysis with prior work, which is essential for establishing baseline performance for both single and ensemble methods considered in our current work. Despite their simulated nature, both datasets include a broad range of anomaly types (e.g., Sybil attacks, DoS, message falsification, lane deviation, protocol violations), which are representative of many real-world threats. This diversity enables a meaningful evaluation of anomaly detection capabilities. Our ensemble learning framework is designed to be modular and adaptable. The models and preprocessing pipelines can be extended to other datasets with minimal modification. We have also released our source code and data processing scripts to facilitate such extensions.

We also note that the most dangerous and challenging anomalies are those that are unknown or novel, and that detecting such samples is a critical goal for robust security systems for autonomous driving applications. While our current study focuses on labeled deviations, we recognize the importance of extending our work to include open-set recognition or out-of-distribution detection methods in future research.

#### 7.4.2. Computational Complexity and Scalability Concerns

Some of the ensemble learning techniques employed in this study, particularly the more sophisticated approaches like Stacking and Blending, exhibit substantial computational overhead and prolonged runtime, as evidenced by the reported results. While these advanced ensemble methods demonstrated superior predictive performance, their computational complexity raises concerns regarding their scalability and applicability in real-time or resource-constrained environments. Autonomous driving systems often require rapid decision-making and low-latency processing, which may be hindered by the computational demands of these ensemble learning techniques. Addressing this limitation might necessitate further optimization, parallelization, or the exploration of more efficient ensemble learning strategies tailored to the requirements of autonomous driving systems.

### 7.5. Explaining Decision-Making of Ensemble Methods

Another thing to note with our current work is that it mainly focuses on ensemble learning for anomaly detection. However, incorporating explainable AI (XAI) [69] to better understand the decision-making of ensemble methods would enhance the applicability of the current work. We emphasize that different ensemble models—particularly complex ones like Stacking and Blending—can behave as black boxes, making it difficult to understand their internal reasoning. For tree-based ensembles (e.g., Random Forest, XGBoost), we suggest using feature importance scores, SHAP (SHapley Additive exPlanations), and LIME (Local Interpretable Model-agnostic Explanations) to visualize and interpret model decisions. For stacked models, we recommend analyzing the meta-learner’s feature contributions and using surrogate models to approximate decision boundaries. We have performed an initial analysis in this paper for feature importance using SHAP for tree-based ensemble methods and sklearn for non-tree-based ensemble methods. One future research direction in this context is to extend our framework to include XAI modules that provide real-time interpretability of anomaly predictions. This will help stakeholders (e.g., engineers, safety analysts) understand why a particular behavior was flagged as anomalous, thereby improving trust and facilitating debugging.

## 8. Conclusions

In this work, we proposed a comprehensive ensemble learning framework to enhance anomaly detection capabilities for autonomous vehicles. Through extensive experiments on two real-world datasets, VeReMi and Sensor, we demonstrated the effectiveness of our ensemble approach in outperforming individual machine learning models across key metrics like accuracy, precision, recall, and F1-score. The ensemble methods, such as Random Forest, Bagging, and Stacking, consistently outperformed single classifiers across various evaluation metrics, including accuracy, precision, recall, and F1-score. The ensemble techniques leverage the strengths of individual models while mitigating their weaknesses, resulting in improved robustness and generalization capabilities. We have obtained an 11% boost in accuracy over single classifiers using ensemble learning models, demonstrating their superiority. Additionally, the analysis revealed the trade-offs between different hyperparameter settings and their impact on model performance, highlighting the importance of careful hyperparameter tuning. Furthermore, this study provides insights into the computational efficiency of the evaluated models and identifies the most time-efficient approaches for practical deployments.

Our hyperparameter analysis revealed intricate trade-offs impacting model performance, underscoring the importance of careful tuning. We also provided insights into the computational efficiency of different models to guide practical deployments. Our feature importance analysis shows the most important features that different ensemble and single models consider when making anomaly detection decisions in our autonomous driving context.

While acknowledging limitations like the need for broader dataset validation, our framework represents a significant step toward robust anomaly detection for safeguarding autonomous vehicles. By synergizing multiple machine learning models, it offers an adaptable solution pivotal to the secure rollout of intelligent transportation systems. By conducting a comprehensive analysis of our ensemble framework’s performance on these diverse datasets, this study elucidated the strengths, limitations, and trade-offs associated with different ensemble learning techniques in the context of anomaly detection for autonomous driving systems.

Several promising avenues exist for extending the current research on ensemble learning for anomaly detection in autonomous vehicles. A natural progression would involve validating the proposed framework’s efficacy on various real-world datasets, encompassing diverse driving scenarios and environmental conditions. Additionally, exploring the integration of advanced feature engineering techniques and unsupervised learning methods could further enhance the discriminative power of the anomaly detection models. Investigating the application of interpretable machine learning frameworks to generate explanations for the ensemble model predictions represents another compelling research direction, facilitating increased transparency and trust in the decision-making process. Furthermore, the development of online learning and adaptive strategies could equip the framework with the capability to dynamically update and refine its models in response to evolving data distributions or emerging attack vectors. Ultimately, real-time implementation and extensive validation in collaboration with domain experts and security analysts would be an essential step toward transitioning this research into practical deployment within autonomous vehicles.

## Figures and Tables

**Table 1:** A comparison between different aspects of our own work and prior relevant works on anomaly detection in autonomous vehicles (including datasets, AI models, methods used, and focus) where N/A means (Not Available).

Related Works	Datasets Used	AI Models	Methods	Focus
Our Work	VeReMi, Sensor datasets	Ensemble Learning (11 ensemble methods)	Ensemble Learning Framework	Robust anomaly detection in AVs and VANETS
Rajendar et al. (2022) [20]	Sensor Data	M-CNN	Anomaly Detection	Sudden abnormalities in AVs
Zekry et al. (2021) [21]	IoT Sensor Data	CNN, Kalman Filtering	Anomaly Detection	Anomalous behaviors in AVs
Alsulami et al. (2022) [22]	AV System Data	LSTM	Anomaly Detection	False Data Injection (FDI) attacks
Prathiba et al. (2023) [23]	Attack Data	Cooperative Analytics	Anomaly Detection	Anomalies in AV networks
Hybrid Deep Anomaly Detection (HDAD) [23]	Shared Sensor Data	Hybrid Deep Learning	Anomaly Detection	Harmful activities in AVs
Time-Series Anomaly Detection [24]	Time-Series Data	N/A	Anomaly Detection	Cyber intrusions, malfunctioning sensors
CNN-based LSTM Model [25]	Signal Data	CNN, LSTM	Anomaly Detection	Anomalous or healthy signals
Scenario Engineering in AVs [26]	N/A	N/A	Scenario Engineering	Trustworthiness, robustness in AVs
Generative AI Models for Scenario Engineering [27]	N/A	Generative AI (Sora)	Scenario Generation	Intelligent vehicle scenarios
Efficient Trajectory Prediction [28]	Argoverse and nuScenes Datasets	Attention, LSTM, GCN, Transformers	Trajectory Prediction	Spatial–temporal information
Reliability-based Path Planning [29]	Deformable Terrain Data	RRT*	Path Planning	Off-road AVs
Advanced Driver Assistance Systems [30]	N/A	CNNs	Various	AV features, object recognition, localization
Driver Activity Recognition System [31]	ImageNet Dataset	CNN (AlexNet, GoogLeNet, ResNet50)	Activity Recognition	Driving activities, distracted behaviors
Semi-supervised K-NN-based Ensemble Learning [11]	Maneuvering Behavior Data	K-NN Ensemble	Semi-supervised Learning	Classifying maneuvering behaviors
Ensemble-based Intrusion Detection System [33]	N/A	Ensemble Learning	Intrusion Detection	Classifying malicious and benign data requests
Ensemble-based Anomaly Detection [13]	Vehicle Data	Ensemble Learning	Anomaly Detection	Identifying potential faults
PelFace [34]	Face Data	Ensemble Learning	Face-based Authentication	Enhancing face-based authentication
Hybrid Ensemble Approach [35]	Radar Data	Random Forest, CNN	Object Classification	Classifying objects using radar data
Data-driven Method for Virtual Merging Scenarios [36]	N/A	N/A	Markov Decision Processes, Game Theory	Modeling vehicle behavior

**Table 2:** Statistics of both VeReMi and Sensor datasets. We emphasize that these anomalous samples are predefined deviations and not necessarily representative of true anomalies in the broader sense.

	VeReMi Dataset	Sensor Dataset
Number of Labels	2 and 6	2
Number of Features	6	10
Dataset Size	993,834	10,000
Training Sample	695,684	7000
Testing Sample	298,150	3000
Normal Samples No.	664,131	5000
Anomalous Samples No.	329,703	5000

**Table 3:** Performance of ensemble learning models for binary classification on VeReMi dataset. RF, Bagging, Avg, W. Avg, Stacking, and Blending show the best accuracy and F1 performance, and the latter four show the best precision. GBM shows the best recall performance for binary classification for VeReMi dataset. The best results are given in bolded text.

Models	Acc	Prec	Rec	F1
RF	**0.80**	0.82	0.91	**0.86**
Bagging	**0.80**	0.82	0.90	**0.86**
AdaBoost	0.73	0.74	0.91	0.82
XGBoost	0.70	0.70	0.96	0.81
CatBoost	0.70	0.70	0.96	0.81
LGBM	0.70	0.71	0.94	0.81
GBM	0.68	0.68	**1.00**	0.81
Avg	**0.80**	**0.83**	0.89	**0.86**
W. Avg	**0.80**	**0.83**	0.89	**0.86**
Stacking	**0.80**	**0.83**	0.89	**0.86**
Blending	**0.80**	**0.83**	0.89	**0.86**

**Table 4:** Performance of single-classifier models for binary classification on VeReMi dataset. DT shows the best overall performance for single-classifier models in binary classification for VeReMi dataset. The best results are given in bolded text.

Models	Acc	Prec	Rec	F1
DT	**0.79**	**0.82**	0.87	**0.84**
MLP	0.66	0.67	**0.96**	0.79
KNN	0.78	**0.82**	0.85	**0.84**
SVM	0.55	0.54	0.66	0.60

**Table 5:** Performance of ensemble classifier models for binary classification on Sensor dataset. CatBoost depicts the best performance over all the metrics in binary classification on Sensor dataset. The best results are given in bolded text.

Models	Acc	Prec	Rec	F1
RF	0.90	0.90	0.98	0.94
Bagging	0.89	0.91	0.96	0.93
AdaBoost	0.99	0.99	**1.00**	0.99
XGBoost	0.98	0.97	**1.00**	0.99
CatBoost	**1.00**	**1.00**	**1.00**	**1.00**
LGBM	0.99	0.99	**1.00**	0.99
GBM	0.86	0.88	0.96	0.92
Avg	0.86	0.86	0.99	0.92
W. Avg	0.89	0.90	0.97	0.93
Stacking	0.98	0.98	0.99	0.99
Blending	0.99	0.99	0.99	0.99

**Table 6:** Performance of single-classifier models for binary classification on Sensor dataset. We observe that MLP is performing the best for accuracy, precision, and F1-score metrics while KNN shows the best performance for recall. The best results are given in bolded text.

Models	Acc	Prec	Rec	F1
DT	0.85	0.89	0.92	0.90
MLP	**0.89**	**0.93**	0.93	**0.93**
KNN	0.84	0.85	**0.97**	0.91
SVM	0.88	0.90	0.95	0.92

**Table 7:** Performance of ensemble classifier models for multiclass classification on VeReMi dataset. Stacking and Blending are performing the best for accuracy, precision, and F1-score while XGBoost and CatBoost show perfect recall for multiclass classification on VeReMi dataset. The best results are given in bolded text.

Models	Acc	Prec	Rec	F1
RF	0.65	**0.79**	0.94	0.86
Bagging	0.66	0.76	0.98	0.86
AdaBoost	0.66	0.69	0.98	0.81
XGBoost	0.67	0.67	**1.00**	0.80
CatBoost	0.67	0.68	**1.00**	0.81
LGBM	0.67	0.68	0.99	0.80
GBM	0.66	0.70	0.97	0.82
Avg	0.65	0.76	0.91	0.83
W. Avg	0.65	0.76	0.91	0.83
Stacking	**0.72**	**0.79**	0.96	**0.87**
Blending	**0.72**	**0.78**	0.96	**0.87**

**Table 8:** Performance of single-classifier models for multiclass classification on VeReMi dataset. MLP and SVM perform the best in accuracy and recall whereas KNN performs the best for precision and F1-score. The best results are given in bolded text.

Models	Acc	Prec	Rec	F1
DT	0.66	0.72	0.97	0.83
MLP	**0.67**	0.67	**1.00**	0.80
KNN	0.65	**0.77**	0.95	**0.85**
SVM	**0.67**	0.67	**1.00**	0.80

**Table 9:** Effect of hyperparameters on VeReMi dataset. We observe that increasing ‘max_depth‘ and ‘lr‘ generally improves ensemble model performance. The best results are given in bolded text.

Metric	RF				Bagging				AdaBoost				XGBoost			
**Hyperparameter**	max_depth		n_estimators		max_depth		n_estimators		max_depth		n_estimators		max_depth		n_estimators	
**Values**	4	50	100	200	4	50	100	200	4	50	100	200	6	50	100	200
Acc	0.67	**0.80**	**0.80**	**0.80**	0.67	**0.80**	**0.80**	**0.80**	0.70	**0.80**	**0.80**	**0.80**	0.69	0.75	0.75	0.75
Prec	0.67	0.83	0.83	0.83	0.67	0.82	0.82	0.82	0.71	**0.84**	0.83	**0.84**	0.70	0.79	0.79	0.79
Rec	**1.00**	0.88	0.88	0.88	**1.00**	0.90	0.90	0.90	0.93	0.86	0.87	0.86	0.97	0.86	0.86	0.86
F-1	0.80	**0.86**	**0.86**	**0.86**	0.80	**0.86**	**0.86**	**0.86**	0.80	0.85	0.85	0.85	0.81	0.82	0.82	0.82
**Metric**	**CatBoost**				**LGBM**				**GBM**							
**Hyperparameter**	depth		lr		num_boost_round		lr		max_depth		n_estimators					
**Values**	6	10	0.5	0.8	100	500	0.01	0.5	10	50	10	50				
Acc	0.70	0.74	0.71	0.74	0.70	0.72	0.70	0.72	0.68	**0.80**	0.68	**0.80**				
Prec	0.70	0.75	0.72	0.75	0.70	0.73	0.70	0.73	0.68	0.83	0.68	0.83				
Rec	0.96	0.91	0.94	0.91	0.96	0.93	0.96	0.93	**1.00**	0.88	**1.00**	0.88				
F-1	0.81	0.82	0.81	0.82	0.81	0.82	0.81	0.82	0.81	0.85	0.81	0.85				

**Table 10:** Effect of hyperparameters on Sensor dataset. We note that increasing ‘max_depth‘ and ‘lr‘ generally improves ensemble model performance. The best results are given in bolded text.

Metric	RF				Bagging				AdaBoost				XGBoost			
**Hyperparameter**	max_depth		n_estimators		max_depth		n_estimators		max_depth		n_estimators		max_depth		n_estimators	
**Values**	4	50	100	200	4	50	100	200	4	50	100	200	6	50	100	200
Acc	0.81	0.82	0.91	0.91	0.83	0.82	0.90	0.90	0.98	0.98	0.84	0.85	0.99	0.98	0.97	0.98
Prec	0.80	0.81	0.91	0.91	0.83	0.84	0.91	0.91	0.98	0.98	0.90	0.90	0.99	0.98	0.97	0.98
Rec	**1.00**	**1.00**	0.98	0.98	0.98	0.96	0.97	0.97	**1.00**	0.99	0.89	0.91	**1.00**	**1.00**	0.99	**1.00**
F-1	0.89	0.90	0.94	0.94	0.90	0.89	0.94	0.94	0.99	0.99	0.90	0.91	0.99	0.99	0.98	0.99
**Metric**	**CatBoost**				**LGBM**				**GBM**							
**Hyperparameter**	lr		depth		lr		num_boost_round		lr		max_depth					
**Values**	0.03	0.5	6	10	0.5	0.8	100	500	0.01	0.5	10	50				
Acc	0.97	**1.00**	**1.00**	0.96	0.99	0.99	0.99	0.99	0.86	0.92	0.92	0.85				
Prec	0.96	**1.00**	**1.00**	0.96	0.99	0.99	0.99	0.99	0.88	0.92	0.92	0.90				
Rec	**1.00**	**1.00**	**1.00**	0.99	**1.00**	**1.00**	**1.00**	**1.00**	0.96	0.98	0.98	0.92				
F-1	0.98	**1.00**	**1.00**	0.97	**1.00**	**1.00**	**1.00**	**1.00**	0.92	0.95	0.95	0.91				

**Table 11:** A summary of our evaluation. Our ensemble learning framework provides higher performance and lower FPR.

Higher Metrics (ACC, PRE, REC, F1)	Sensor	VeReMi	Overall
Best Setup	Ensemble	Ensemble	Ensemble
Best Models	CatBoost, LGBM, Stacking, Blending, AdaBoost	RF, Bagging, Avg, W. Avg, Stacking, Blending, LGBM, DT	Blending, Stacking, CatBoost, DT, LGBM, RF, AdaBoost
**Lower FPR (%)**	**Sensor**	**VeReMi**	**Overall**
Best Setup	Ensemble	Ensemble/Single	Ensemble
Best Models	RF, AdaBoost, Stacking, CatBoost, Bagging, LGBM, MLP	RF, AdaBoost, Stacking, DT, KNN	RF, AdaBoost, Stacking
**Runtime**	**Sensor**	**VeReMi**	**Overall**
Fastest Models (less than 1 min in all level variants)	RF, Bagging, AdaBoost, XGBoost, CatBoost, LGBM, GBM, DT, MLP, KNN, SVM	LGBM, XGBoost, AdaBoost, DT, KNN	AdaBoost, XGBoost, LGBM, DT, KNN
Average Models (1 and 10 min in all level variants)	Avg, W. Avg, Stacking, Blending	RF, CatBoost, Bagging, GBM, SVM	Avg, W. Avg, GBM, Stacking, Blending, SVM
Slowest Models (more than 10 min in all level variants)	-	Stacking, Blending, Avg, W. Avg, MLP	Stacking, Blending, Avg, W. Avg, MLP

## Data Availability

We adhere to the data availability policy outlined by MDPI journals. The data supporting the findings of this study are available in the public repository at the following URL. The used datasets are available at https://github.com/Nazat28/Ensemble-Models-for-Classification-on-Autonomous-Vehicle-Dataset (accessed on 1 August 2025).

## Abbreviations

The following abbreviations are used in this manuscript:

AVs	Autonomous Vehicles
VANET	Vehicular Ad Hoc Network
KNN	K-nearest Neighbors
ADB	Aggressive Driving Behavior
RF	Random Forest
AdaBoost	Adaptive Boosting
XGBoost	EXtreme Gradient Boosting
CatBoost	Categorical Boosting
LGBM	Light Gradient Boosting Machine
Avg	Averaging
W. Avg	Weighted Average
Blend	Blending
M-CNN	Modified-Convolutional Neural Network
MLP	Multilayer Perceptron
LSTM	Long-Short Term Memory
SVM	Support Vector Machine
DT	Decision Tree
FDI	False Data Injection
OBU	Onboard Unit

## Appendix A. AI Models and Hyper-Parameters

We now provide the hyperparameters of different AI models considered in this work.

### Appendix A.1. AI Models and Hyperparameters Details

#### Appendix A.1.1. Base Models

First, we provide details of the base models used in this work.

**Decision Tree (DT)**: The best hyperparameter choice for DT model was when the criterion was set to “gini” for measuring impurity and the max depth of the tree was 50, which means the number of nodes from the root node to the last leaf node was 50. The minimum number of samples required to be present in a leaf node was 4 (min_samples_leaf=4), and the rest of the hyperparameters were set as default.

**K-nearest Neighbors (KNN)**: All the hyperparameters used by KNN were set to their default values.

**Support Vector Machine (SVM)**: For the SVM AI classifier, we set the regularization parameter to 1 (C = 1). The kernel function was set to the radial basis function (RBF). The kernel coefficient to control the decision boundary was set to “auto”. All other hyperparameters were set to default.

**Multilayer Perceptron (MLP)**: The next used classifier was MLP. We used the following setup for the MLP classifier: the hidden_layer_sizes was set to (100, 50), implying a neural network architecture with two hidden layers comprising 100 and 50 neurons, respectively. The max_iter parameter is set to 500, governing the maximum number of iterations or epochs for training. The activation function for the hidden layers is defined as ‘relu’, short for Rectified Linear Unit. The optimization algorithm chosen is ‘adam’, an extension of stochastic gradient descent. Lastly, the random_state is set to 42 to ensure reproducibility of results through consistent random initialization.

#### Appendix A.1.2. Ensemble Methods

Second, we lay out the main details of ensemble methods.

**Random Forest (RF):** The next classifier used to detect anomalous samples in the VANET system was the Random Forest (RF). The hyperparameters we used for this classifier are as follows: n_estimators (a parameter that signifies the number of trees used) was set to the value of 100, the maximum tree depth was set to the value of 50, the minimum number of samples required to separate internal node was set to the value of 4, and the rest were used as provided by default.

**AdaBoost (ADA)**: The next classifier used in this study was AdaBoost. We used the sklearn library for that classifier. The parameter configuration for this classifier is as follows: the maximum number of estimators at which boosting will be completed was set to a value of 50, the max_depth was set to 10, and the base estimator from which the boosted ensemble is built was set to Decision_Tree_Classifier.

**LightGBM (LGBM)**: In LightGBM model training, the hyperparameters are configured as follows: a learning rate of 0.80 controls optimization steps, employing a boosting type of ’gbdt’ (Gradient Boosting Decision Trees). The objective function is set to ’binary’ for binary classification tasks, with evaluation using the ’binary_logloss’ metric. Trees are constrained to a maximum depth of 10.

**Extreme Gradient Boost (XGB)**: The next classifier is XGB. We used the xgboost Python library for that classifier. For the XGBoost classifier instantiation, the hyperparameters are succinctly outlined as follows: The objective function is set to ’binary:logistic’, indicating binary classification with logistic regression as the base learner. Evaluation metric is specified as ’logloss’, assessing the model’s performance using logarithmic loss.

**CatBoost (CAT)**: We used the catboost library from Python. All the hyperparameters we set for CatBoost were the default values.

**Average**: The next classifier is Average. This method is a simple stacking method that checks each model decision and averages its decision to decide its outcome.

**Weighted Average**: The next classifier is Weighted Average. This method is a simple stacking method that checks each model and averages its decision to decide its outcome, but each model has a different weight based on its importance. We have provided the full details of the weights in our shared Github link (shared in the Section 1).

**Bagging**: The next classifier set is a class of ensemble methods called Bagging. We used the sklearn library for that classifier. This method divides the dataset into subsets with replacements, and these different subsets are the input data to the models used. Then the outcomes are combined to reach a combined decision.

**Stacking**: The next classifier set is a class of ensemble methods called Stacking. We used the sklearn.ensemble library for that classifier. This method stacks the models’ decisions and uses their outcomes to create a new dataset. Then, models and ensemble methods are applied again to see the outcome of the stacking model.
